# Gene Co-expression Analysis Indicates Potential Pathways and Regulators of Beef Tenderness in Nellore Cattle

**DOI:** 10.3389/fgene.2018.00441

**Published:** 2018-10-05

**Authors:** Tássia Mangetti Gonçalves, Luciana Correia de Almeida Regitano, James E. Koltes, Aline Silva Mello Cesar, Sónia Cristina da Silva Andrade, Gerson Barreto Mourão, Gustavo Gasparin, Gabriel Costa Monteiro Moreira, Elyn Fritz-Waters, James M. Reecy, Luiz Lehmann Coutinho

**Affiliations:** ^1^Department of Animal Science, University of São Paulo, Piracicaba, Brazil; ^2^Embrapa Southeast-Cattle Research Center, São Carlos, Brazil; ^3^Department of Animal Science, Iowa State University, Ames, IA, United States; ^4^Department of Genetics and Evolutionary Biology, University of São Paulo, São Paulo, Brazil

**Keywords:** meat, bovine, RNA-Seq, transcriptome, networks, microRNA

## Abstract

Beef tenderness, a complex trait affected by many factors, is economically important to beef quality, industry, and consumer’s palatability. In this study, RNA-Seq was used in network analysis to better understand the biological processes that lead to differences in beef tenderness. Skeletal muscle transcriptional profiles from 24 Nellore steers, selected by extreme estimated breeding values (EBVs) for shear force after 14 days of aging, were analyzed and 22 differentially expressed transcripts were identified. Among these were genes encoding ribosomal proteins, glutathione transporter ATP-binding cassette, sub-family C (CFTR/MRP), member 4 (*ABCC4*), and synaptotagmin IV (*SYT4*). Complementary co-expression analyses using Partial Correlation with Information Theory (PCIT), Phenotypic Impact Factor (PIF) and the Regulatory Impact Factor (RIF) methods identified candidate regulators and related pathways. The PCIT analysis identified ubiquitin specific peptidase 2 (*USP2*), growth factor receptor-bound protein 10 (*GBR10*), anoctamin 1 (*ANO1*), and transmembrane BAX inhibitor motif containing 4 (*TMBIM4*) as the most differentially hubbed (DH) transcripts. The transcripts that had a significant correlation with *USP2*, *GBR10*, *ANO1*, and *TMBIM4* enriched for proteasome KEGG pathway. RIF analysis identified microRNAs as candidate regulators of variation in tenderness, including *bta-mir-133a-2* and *bta-mir-22*. Both microRNAs have target genes present in the calcium signaling pathway and apoptosis. PIF analysis identified myoglobin (*MB*), enolase 3 (*ENO3*), and carbonic anhydrase 3 (*CA3*) as potentially having fundamental roles in tenderness. Pathways identified in our study impacted in beef tenderness included: calcium signaling, apoptosis, and proteolysis. These findings underscore some of the complex molecular mechanisms that control beef tenderness in Nellore cattle.

## Introduction

Meat tenderness is an important beef palatability trait for consumers, and most of the biological mechanisms involved in this trait are not completely understood (Ouali et al., 2006). Beef tenderness is commonly measured by SF and is influenced by genetic factors, environment and genotype-environment interaction (Koch et al., 1982; Wheeler et al., 2005). The average heritability for beef tenderness is about 0.3, corroborating that genetics have a reasonable contribution for this trait (Koch et al., 1982). Unfortunately, tenderness and other phenotypes associated with meat quality such as taste and color, are difficult to measure directly, and can only be determined after an animal is slaughtered (Gonçalves, 2015; Mateescu et al., 2017).

For complex traits such as beef tenderness, next-generation sequencing (NGS) technologies have been applied in transcriptomic analyses to identify DE genes and biological pathways potentially involved with meat quality phenotypes (Metzker, 2010). However, biological interpretation of RNA-Seq remains a challenge (Barabasi and Oltvai, 2004; de la Fuente, 2010). Co-expression analysis provides a powerful approach to integrate the transcripts relationship into different pathways based on phenotypic variation (Reverter et al., 2006; Reverter and Chan, 2008). The PCIT, RIF, and PIF algorithms determine the changes in correlation or wiring of a network between treatments or extreme levels of phenotypes. PCIT identifies significant partial correlations among pairs of genes and can be used to quantify differential connectivity as well as the strength of a connection to a hub gene (Reverter et al., 2006; Reverter and Chan, 2008). PIF and RIF analyses are based on the approach of differential wiring or differential correlation, which is the difference in co-expression between a certain pair of genes under two different situations. More specifically, PIF is the average expression of two conditions combined, multiplied by the DE value of genes. PIF scores allow the variability and differential expression of a gene to be prioritized in determining its importance in relation to differences in phenotypic variation (Reverter et al., 2010). RIF is calculated from the cumulative differential wiring of each regulator compared to the target DE genes and weighted for PIF (Hudson et al., 2009). The RIF algorithm can identify genes that may not show differential expression but still alter the molecular “wiring” of a pathway or act as biomarkers of phenotypic variation. For instance, myostatin was identified as a regulator in a study of gene expression was conducted between two breeds of cattle, Piedmontese; which is double-muscled due to a mutation in the myostatin gene, and Wagyu, which do not contain the mutation. The key regulator of muscle growth, myostatin, was not DE in animals carrying the mutation, but this gene was differentially wired based on the RIF analysis (Hudson et al., 2009).

Previous gene co-expression networks analysis allowed the integration of regulatory processes, which affected gene expression level of the studied system (Reverter et al., 2006). Complex networks of gene interactions influence meat quality, including beef tenderness (Mateescu et al., 2017). Understanding the processes involved in postmortem traits impacting meat quality is critical to elucidate the mechanisms involved in the conversion of muscle to meat (Ponsuksili et al., 2013). The discovery of potential regulators by gene co-expression analysis includes small RNAs. MiRNAs are small non-coding RNAs that are highly conserved among mammals and can regulate translation by binding to the 3′-UTR of target mRNAs. They play a role in many biological processes including myogenesis and hypertrophy (Wang et al., 2014). In muscle, miRNAs can regulate myoblast proliferation and differentiation and may control gene expression related to the variation of beef tenderness during stress (Zhao et al., 2012a). The identification of miRNAs enriched in skeletal muscle can enhance the understanding of its biology and function (Wang et al., 2014). Thus, gene co-expression analysis can be used to possibly identify miRNAs that regulate the skeletal muscle development.

The objective of this study was to identify DE genes associated with beef tenderness in skeletal muscle (LD) from Nellore cattle selected for extreme estimated breeding values (EBVs) for SF after 14 days of aging. A secondary goal was to unveil possible mechanisms associated with biological processes and variation in beef tenderness at the pathway level. The final goal was to explore regulators involved in beef tenderness variation using co-expression methods (PCIT, PIF, and RIF) in order to increase our understanding of genes and pathways involved in the variation of beef tenderness.

## Materials and Methods

### Animals and Phenotypic Data

The Institutional Animal Care and Use Committee (IACUC) Guidelines from the Brazilian Agricultural Research Corporation – EMBRAPA approved all experimental procedures involving steers prior to conducting this study (Macroprograma 1, 01/2005). The animals selected for RNA-Seq analysis came from a larger project where meat quality traits were evaluated (Tizioto et al., 2013a). A total of 310 Nellore steers sired by 33 unrelated Nellore bulls were studied. The sires were selected based on the national summary of Nellore cattle to represent the main breeding lineages used in Brazil. Steers were raised in feedlots with identical nutritional rations and handling conditions until slaughter at an average age of 25 months as previously reported (Cesar et al., 2014). Samples from skeletal muscle (LD) located between the 12th and 13th ribs were collected at two-time points: at slaughter for RNA sequencing analysis, and 24 h after slaughter for SF analysis and aging.

The beef tenderness was represented by the SF measured from the steaks with 2.54 cm of thickness. The SF was obtained by using a TA XT2i (Stable Micro Systems Ltd., Surrey, United Kingdom) texture analyzer coupled to a Warner-Bratzler blade, at 1.016 mm thickness (Wheeler et al., 1997). Measurements of SF were made after aging in a 2°C chamber for 24 h at 7 and 14 days after slaughter (Tizioto et al., 2013a). The measurements at 14 days of aging confirmed the results of tenderness identified at 7 days of aging and were used as the SF phenotype.

### Statistical Analysis of Shear Force

A goal of the larger overarching meat quality project in Nellore was to determine the impact of additive genetic control on meat quality traits and to develop genetic breeding tools to improve these traits in Nellore cattle. For this reason, animals were ranked based on the EBV, instead of the phenotypic value. The EBV represents the animal’s predicted genetic merit, half of which is inherited by its progeny to impact their performance. The benefits of using EBV are the ability (1) to make full use of information from all relatives; (2) to account for fixed environmental effects so that animals can be compared across groups, giving wider scope for selection by focusing on genetic effects (Sosnicki and Newman, 2010). Therefore, EBVs were used to select the extreme groups of animals for RNA-Seq, PCIT, PIF, and RIF analyses.

Prior to RNA-Seq analysis, EBVSF were obtained based on the SF performance of the half-sib data using standard best linear unbiased prediction (BLUP) procedures under an animal model performed using the mixed procedure of SAS (Henderson, 1973; Saxton and SAS Institute, 2004; Mrode, 2014). Animals were ranked based on the EBVSF at 14 days of aging in order to adjust for fixed effects that could interfere with phenotypic measurements. Selected animals were divided into two groups with extreme values: highest (H) values of EBVSF (*n* = 11) and lowest (L) values of EBVSF (*n* = 13). The mixed model used to estimate breeding values contained a numerator coefficient matrix of relationship (A) containing 284 animals. The basic model equation in matrix notation was as follows:

γ=Xβ+Zu+ε

Where the γ (dependent variable) is the phenotype SF phenotype in kgf/cm^2^, and *X* is the incidence matrix of fixed effects associated with the vector of fixed effectsβ. Fixed effects included contemporary group (based on slaughter group and birth season) and age, which was used as a linear covariate. The incidence matrix of random animal effects in *u*, where *u* ∼ N (0, Aσ_a_^2^) for SF at 14 days of aging was represented by Z. The random animal effect was included to estimate the additive genetic effect. The ε is the vector of random residuals, normally and independently distributed as ε∼ (NID) (0, Iσ_e_^2^). Variance component estimation was conducted by restricted maximum likelihood (REML) methodology (Patterson and Thompson, 1971).

### RNA-Seq Analysis

Total RNA of 24 LD muscle samples (100 mg) were extracted with Trizol reagent (Thermo Fisher Scientific, Carlsbad, CA, United States). The RNA was quantified using a spectrophotometer (NanoDrop 200 - Thermo Fisher Scientific, Wilmington, DE, United States). RNA Integrity Number (RIN) was verified using a 1% agarose gel and a Bioanalyzer 2100 (Agilent Technologies, Santa Clara, CA, United States). All samples had RIN values greater than seven.

Libraries were prepared using the TruSeq SBS Kit v3-HS (200 cycles). The average cDNA library size was estimated using an Agilent Bioanalyzer 2100 (Agilent Technologies, Santa Clara, CA, United States) and quantified using quantitative PCR with the KAPA Library Quantification kit (KAPA Biosystems, Foster City, CA, United States). Samples were diluted to 17 pM and clustered on the sequencing flowcell by cBOT (Illumina, San Diego, CA, United States) using the TruSeq paired-end (PE) Cluster Kit v3-cBOT-HS kit. Libraries were barcoded, multiplexed, and sequenced in seven lanes on a HiScanSQ (Illumina, San Diego, CA, United States) to generate 100 nt-long paired-end reads. These procedures were performed at the Center for Functional Genomics (USP, Campus “Luiz de Queiroz”, SP, Brazil).

Sequence quality was evaluated with FastQC (Andrews, 2010). Phred quality score of the reads was assessed using the software Seqyclean (Zhbannikov et al., 2013). Reads with nucleotide quality score < 26 and reads length < 65 bp were discarded. Potential vector and adaptor sequences were compared to the UniVec database to remove possible artificial sequence contaminants. Sequence reads were aligned to the UMD3.1 *Bos taurus* masked reference genome (Ensembl release 80) using TopHat 2.0.10 (Trapnell et al., 2009; Zimin et al., 2009). Reads with more than one mismatch or non-unique mapping were discarded. A reference-guided assembly of transcripts was performed using Cufflinks v2.1.1 to identify novel transcripts (Trapnell et al., 2010). Read counts for each transcript were quantified with HTSeq v0.5.4p2, and used for differential expression analysis (Anders and Huber, 2010).

### Differential Expression Analysis

Prior to the DE analysis, raw count data was filtered for zero (non-expressed) and low expression to ensure transcripts were expressed across the samples (Lund et al., 2012). First, all transcripts with zero counts were removed from all samples as these genes were assumed to be non-expressed in skeletal muscle. Second, all transcripts were required to have at least two reads to remove very lowly expressed genes. Finally, transcripts were required to have non-zero read counts in 1/5 of all samples to remove transcripts that may be quantified in error. In this study, transcripts with no reads in six or more samples were dropped from the analysis. Read count data was normalized for differences in library size using the 75% Quartile method to account for differences in RNA quantity and other library effects (Bullard et al., 2010).

The R package QuasiSeq, based on quasi-likelihood methods added to the edgeR software, was used to estimate the read count dispersion for each gene (Lund et al., 2012). The use of QuasiSeq for DE analysis allowed fixed effects to be added into the original generalized linear model as well as a variable to account for over-dispersion to reduce the error in modeling differences in gene expression. An ANOVA model was conducted in SAS to determine what variables might be important to the differential expression analysis. Final age nested within year, contemporary group (slaughter group, origin, and birth season) and lane of sequencing within flow cell were included as fixed effects in the QuasiSeq gene expression model since they were significant in the SAS analysis (*P*-value < 0.05). The QLSpline effect fitted a quasi-negative binomial distribution to account for over-dispersion in the count data (Lund et al., 2012). This approach identified DE transcripts as a function of SF at 14 days of aging. High and low groups were defined for EBVSF at 14 days of aging and used to select the animals, while the phenotype for QuasiSeq analysis was SF at 14 days of aging. The groups H and L for EBVSF at 14 days of aging were used to discuss the association of transcripts with the SF phenotype. The direction of expression will be up or down-regulated based on the b values, and the average of the normalized counts are enough to represent the direction of expression. The q-value methodology was used to control the false discovery rate (FDR) at 10% (Storey and Tibshirani, 2003).

### Annotation, Functional Analysis, and miRNA Prediction

The Ensembl 80 annotation file used herein contains entries representing both protein coding genes and types of RNAs such as rRNA, snoRNA, snRNA, miRNA, lincRNA, and pseudogenes. When annotation was unavailable for genes identified as DE, several approaches were used to obtain annotations in this study. Novel transcripts identified by Cufflinks were annotated using the Genome2Seq and GOanna tools at AgBase (McCarthy et al., 2010; Trapnell et al., 2010). Uncharacterized transcripts were annotated by searching for orthologous genes at BioMart using Ensembl v80 database (Cunningham et al., 2014). CUFFs transcripts (short transcripts assembled by Cufflinks or transfrags in GTF format) were annotated by the best sequence match generated by the NCBI Basic Local Alignment Search Tool (BLAST) (Altschul et al., 1997).

Functional enrichment analysis is a very effective way to provide biological knowledge to a large dataset. The functional analysis of curated gene ontology terms was conducted with the Database for Annotation, Visualization and Integrated Discovery (DAVID) v6.7 tool to identify Gene Ontology (GO) terms and KEGG pathways with significant enrichment scores (Huang et al., 2008). The Benjamini and Hochberg correction was used to account for multiple testing (FDR < 10%) in this process (Benjamini and Hochberg, 1995).

Standard RNA-Seq protocols that capture poly-A RNAs will, as an added benefit, capture the expression levels of miRNA precursors (pre-miRNAs) as they are initially polyadenylated. It was not the main objective of this study to identify miRNAs, but since it was possible to retain longer pre-miRNAs, we were able to subsequently identify predicted target genes and functions associated with the targets. Pre-miRNAs identified in this study exhibited transcript lengths higher than 65 bp and were reported by the name of the annotated miRNA according to Ensembl database and miRBase. A miRNA target prediction method was used to allow the exploration of miRNA targets potentially regulated by a miRNA (Betel et al., 2008). Transcript targets were predicted with Miranda v3.3a software for miRNAs of interest using the Target Scanning Algorithm (Enright et al., 2003; Betel et al., 2008). Predicted transcript targets of each miRNA were analyzed for over-representation of pathway annotations using Ingenuity Pathway Analysis (IPA) (Enright et al., 2003).

### PCIT, PIF, and RIF Co-expression Analysis

A modified version of PCIT was used to calculate the direct and partial correlations of all possible pair-wise transcript interactions (Reverter and Chan, 2008; Koesterke et al., 2013). The significance of these pair-wise interactions was determined using an information theory approach (Reverter and Chan, 2008). In order to account for gene length to compare transcripts to one another, gene expression values were transformed to fragment per kilobase per million mapped reads (FPKM) values. The FPKM values were corrected for the same covariates used for the QuasiSeq model to create residual FPKMs for each transcript used in the PCIT analysis. The differential hubbing or differential connectivity is the difference in the significant connections (i.e., correlations) of a gene between the two experimental conditions under study. This is computed by subtracting the significant connections a gene has in the two conditions (Hudson et al., 2009). Only significant partial correlation values ≥ 0.90 were used in the DH analysis to determine differential connectivity of hub genes. Thus, the DH analysis considered only the hub genes. DH was computed as the difference of significant connections of a transcript between the H versus the L group (Reverter and Chan, 2008; Hudson et al., 2009; Koesterke et al., 2013). For each DH transcript, a list of all positively and all negatively correlated transcripts was generated. The transcripts with a significant correlation with DH transcripts are called DH transcript targets, and they were tested by gene set enrichment analysis to determine if DH targets were biologically meaningful based on the pathways impacted. These data were analyzed using DAVID to identify over-represented GO terms and KEGG pathways, where the FDR was controlled using Benjamini and Hochberg methodology (FDR < 10%) (Benjamini and Hochberg, 1995). The PCIT results were visualized by BioLayout software (Enright and Ouzounis, 2001).

In addition, a modified RIF analysis was used to identify changes in the correlation of a network between the H and L SF groups (Yang et al., 2015). Instead of limiting possible regulators to transcription factors, all transcripts (including those that mapped on miRNA entries present in Ensembl v80 annotation file) were tested as possible regulators in this analysis. To determine the importance of each DE transcript with respect to phenotype, PIF was also calculated for each transcript. RIF1 and RIF2 scores were calculated as described by Reverter et al. (2010) and modified to consider all transcripts as regulators as described by Yang et al. (2015) using residual FPKM gene expression values, correlation values from PCIT (≥0.90) and PIF values for all transcripts (Reverter et al., 2010; Yang et al., 2015).

## Results

Differential expression analysis identified 22 DE transcripts (*q*-value < 0.10) for SF at 14 days of aging. The top DH transcripts identified by PCIT analysis and the functional analyses performed on the correlated transcripts were associated with the proteasome KEGG pathway. Two miRNAs *bta-mir-133a-2* and *bta-mir-22* were identified among the top regulatory candidates by RIF analysis. The myoglobin (*MB*), enolase 3 (*ENO3*), and carbonic anhydrase 3 (*CA3*) were top transcripts identified by PIF analysis.

### Phenotypic and Sequencing Results

Previously, as part of the larger phenotypic study of this population, the average SF at 24 h, seven and 14 days were estimated at 8.70, 5.93, and 4.56 kgf/cm^2^, respectively (Tizioto et al., 2013a). In order to focus on the genetic mechanisms that influence variation in SF at 14 days of aging, animals with high and low EBVSF were selected for RNA-Seq analysis. The extreme animals selected for this study had average SF values of 6.52 kgf/cm^2^ for the H group and 2.73 kgf/cm^2^ for the L group at 14 days of aging. The selected animals were among the 5% most extreme for SF at 14 days of aging relative to the full population. **Table 1** provides the phenotypes for each group of individuals selected for RNA-Seq, the average measurements of SF at different levels of aging, the EBV values, and the EBV accuracies. The phenotypes were consistent with a normal aging process, with a reduction in SF from 24 h to 14 days for both the H and L groups (**Table 1**).

**Table 1:** Average shear force (kgf/cm^2^), EBVSF values and corresponding accuracies calculated for the low (L) group and high (H) groups at seven and 14 days of aging.

Group	Shear Force			EBV^2^		EBV Accuracy^3^	
Time of aging	24h^1^	7 days	14 days	7 days	14 days	7 days	14 days
High	9.24	7.54	6.52	0.62	0.73	0.34	0.34
Low	8.06	3.18	2.73	−0.65	−0.5	0.33	0.33

*^1^There was no BLUP analysis 24 h after harvest. ^2^EBV, Estimated breeding values. ^3^EBV accuracy, how well the estimate represents the true breeding value.*

On average, 31 million sequence reads were generated per sample from sequencing, and Seqyclean removed 25% of these reads. Approximately 19 million reads were aligned to the 3.1 UMD *Bos taurus* genome per sample. On average, 84% of the reads were mapped, and of these, 77% aligned uniquely (reads that aligned once in the genome). Read mapping statistics, including the total number of reads mapped and the percentage of reads mapped uniquely to the genome, are provided in **Supplementary Table A**.

### Identification of Differentially Expressed Transcripts

A total of 15,693 annotated transcripts were identified after data filtering, including 1,743 novel transcripts. From the 22 transcripts identified as DE (*q*-value < 0.10) by QuasiSeq, eight had higher expression in the H group, and 14 transcripts had higher expression in the L group. All the DE transcripts were annotated, except for one where no annotation could be identified (**Table 2**). A complete list of the relative expression levels is presented in the **Supplementary Table B**.

**Table 2:** Differentially expressed transcripts obtained between H and L groups for EBVSF at 14 days of aging in Nellore steers with *q*-value < 0.10.

Transcript ID	Gene symbol	Description	*q*-value^1^
**DE transcripts with higher expression in the H group**			
ENSBTAT00000002357	*SYT4*	Synaptotagmin IV	7.78E-07
ENSBTAT00000031443	*DCSTAMP*	Dendrocyte expressed seven transmembrane protein	1.16E-06
ENSBTAT00000028731	*PRSS2*	Protease, serine, 2 (trypsin 2)	5.77E-06
ENSBTAT00000016190	*APOL3*	Apolipoprotein L3	3.80E-03
ENSBTAT00000059671	*SNORD113*	Small nucleolar RNA SNORD113/SNORD114 family	3.90E-03
CUFF.14879	*MT-ATP6*	Mitochondrially encoded ATP synthase 6	7.41E-02
ENSBTAT00000022021	*EME2*	Endonuclease EME2-like	8.18E-02
ENSBTAT00000011782	*KIAA1456*	Putative methyltransferase KIAA1456 homolog	9.73E-02
**DE transcripts with higher expression in the L group**			
ENSBTAT00000064379	*RPL9*	60S ribosomal protein L9	4.82E-05
ENSBTAT00000012115	*RPS15*	40S ribosomal protein S15	6.39E-05
CUFF.45691	*AP1M1-GOLGI*	Adaptor-related protein complex 1, mu 1 subunit	6.43E-05
ENSBTAT00000050149	*RPL36*	60S ribosomal protein L36	1.00E-04
CUFF.1369	*ACOT11*	Acyl-CoA thioesterase 11	2.00E-04
ENSBTAT00000060649	*7SK*	7SK RNA	5.00E-04
ENSBTAT00000064020	*RPL31*	60S ribosomal protein L31	5.00E-04
ENSBTAT00000027356	*RPS14*	40S ribosomal protein S14	1.00E-03
ENSBTAT00000010182	*ABCC4*	ATP-binding cassette, sub-family C (CFTR/MRP), member 4	2.60E-03
CUFF.20989	*PGM2L1*	Phosphoglucomutase 2-like 1	3.40E-03
CUFF.14892	Unannotated^2^	Unannotated	4.70E-03
ENSBTAT00000030625	*EEF1A1*	Eukaryotic translation elongation factor 1 alpha 1	2.75E-02
CUFF.2483	*JAM2*	Junctional adhesion molecule B	6.73E-02
ENSBTAT00000013010	*B4GALNT2*	Beta-1,4-*N*-acetyl-galactosaminyl transferase 2	7.27E-02

*^1^q-value methodology was used to control the false discovery rate (FDR) at 10%. ^2^CUFF.14892 was not able to be annotated using Genbank and Ensembl databases.*

The low number of DE transcripts may be due to the complex statistical model used to perform the analysis, including many variables. For this reason, there was no significantly enriched GO term and KEGG pathway from the DE transcripts based on DAVID analysis (FDR adjusted *P*-value < 0.1).

### Hub Transcripts Identified With PCIT

PCIT analysis identified transcripts that may be acting as regulators of pathways impacting tenderness. The DH analysis identified correlated transcripts with an absolute correlation ≥ 0.9. **Table 3** reports the top 10 negative and positive DH transcripts when comparing the H group relative to L group.

**Table 3:** Top 10 negatively and positively differentially hubbed (DH) transcripts.

Transcript ID	Gene symbol	Description	DH
**Negative hub transcripts**			
ENSBTAT00000052414	*MEX3C*	Mex-3 RNA binding family member C	−1412
ENSBTAT00000025085	*PTPN3*	Protein tyrosine phosphatase, non-receptor type 3	−1276
ENSBTAT00000002293	*TRIM45*	Tripartite motif containing 45	−1035
ENSBTAT00000012857	*USP2*	Ubiquitin specific peptidase 2	−1021
ENSBTAT00000045760	*RTP4*	Receptor (chemosensory) transporter protein 4	−1013
ENSBTAT00000017090	*NT5C2*	5’-nucleotidase, cytosolic II	−982
CUFF.37260	*GRB10*	Growth factor receptor-bound protein 10	−959
ENSBTAT00000014551	*HECTD4*	HECT domain containing E3 ubiquitin protein ligase 4	−953
ENSBTAT00000021617	*AKTIP*	AKT interacting protein	−923
ENSBTAT00000002122	*ALPK3*	Alpha-kinase 3	−922
**Positive hub transcripts**			
ENSBTAT00000008243	*TMBIM4*	Transmembrane BAX inhibitor motif containing 4	3975
ENSBTAT00000028368	*STXBP6*	Syntaxin binding protein 6 (amisyn)	3832
ENSBTAT00000043719	*XRCC2*	X-ray repair complementing defective repair in Chinese hamster cells 2	3805
ENSBTAT00000024342	*TMEM150A*	Transmembrane protein 150A	3791
ENSBTAT00000031479	*HAUS6*	HAUS augmin-like complex, subunit 6	3729
ENSBTAT00000009560	*EIF2B1*	Eukaryotic translation initiation factor 2B, subunit 1 alpha, 26 kDa	3709
ENSBTAT00000004547	*ENPP4*	Ectonucleotide pyrophosphatase/phosphodiesterase 4 (putative)	3692
ENSBTAT00000013189	*ANO1*	Anoctamin 1, calcium activated chloride channel	3670
ENSBTAT00000012410	*SLC25A44*	Solute carrier family 25, member 44	3663
ENSBTAT00000018878	*CDK5RAP3*	CDK5 regulatory subunit associated protein 3	3653

Functional enrichment analysis was performed on the targets of DH transcripts to determine their biological relevance. Each of the top DH transcripts, independent of their DH value, exhibited positively and negatively correlated transcript targets that were enriched for GO terms by DAVID tools (FDR adjusted *P*-value < 0.1). The **Supplementary Tables C–V** present the most significant (FDR adjusted *P*-value < 0.1) enrichment results, including GO terms of biological process (BP), molecular function (MF), cellular component (CC), and KEGG pathway information for all negatively and all positively correlated transcript targets of the DH transcripts presented in **Table 3**.

**FIGURE 1:**
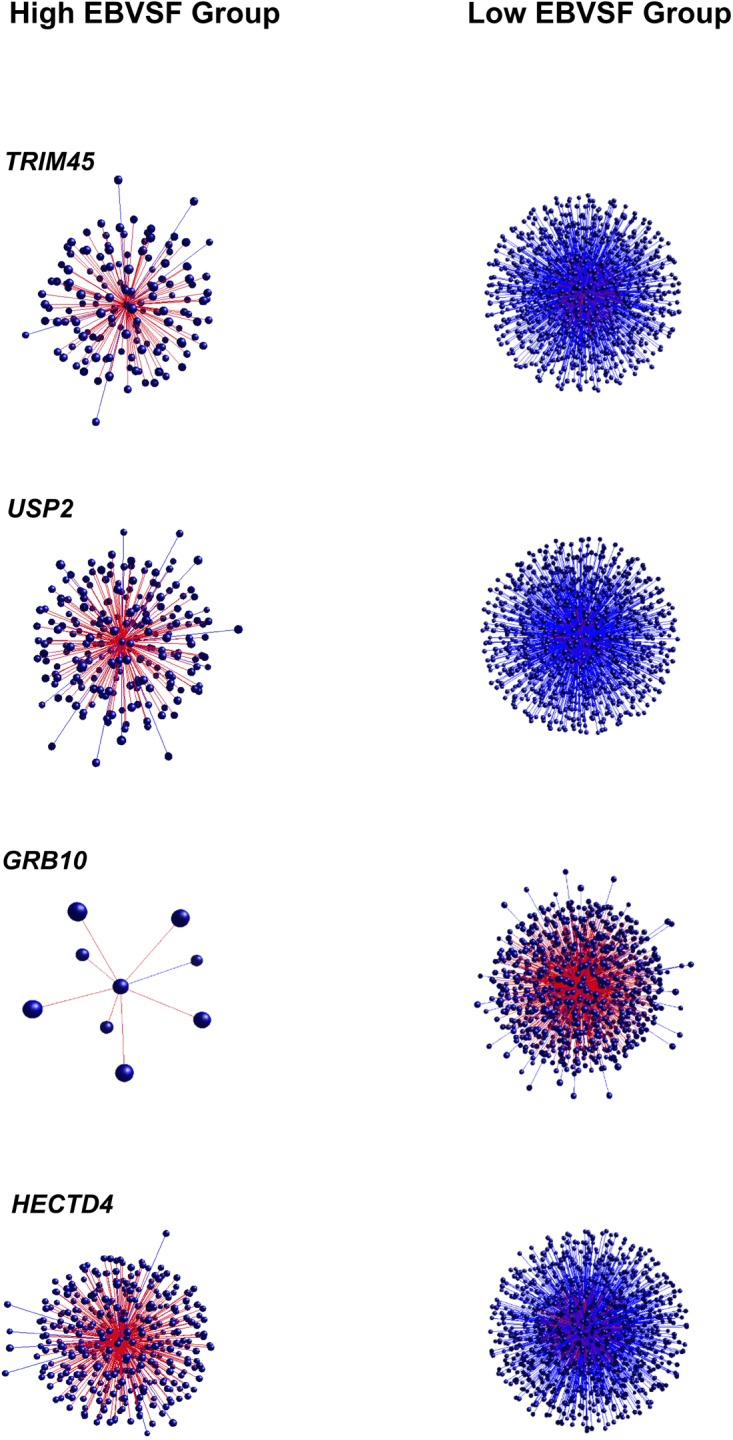
Negative DH between the H and L groups for EBVSF at 14 days of aging. The center spot represents a hub transcript with a high value of DH, red lines represent positive DH and blue lines represent negative DH. All lines represent significant partial correlations ≥ 0.90 at *p*-value < 0.05 based on the information theory approach, which accounts for all comparisons in the analysis.

**FIGURE 2:**
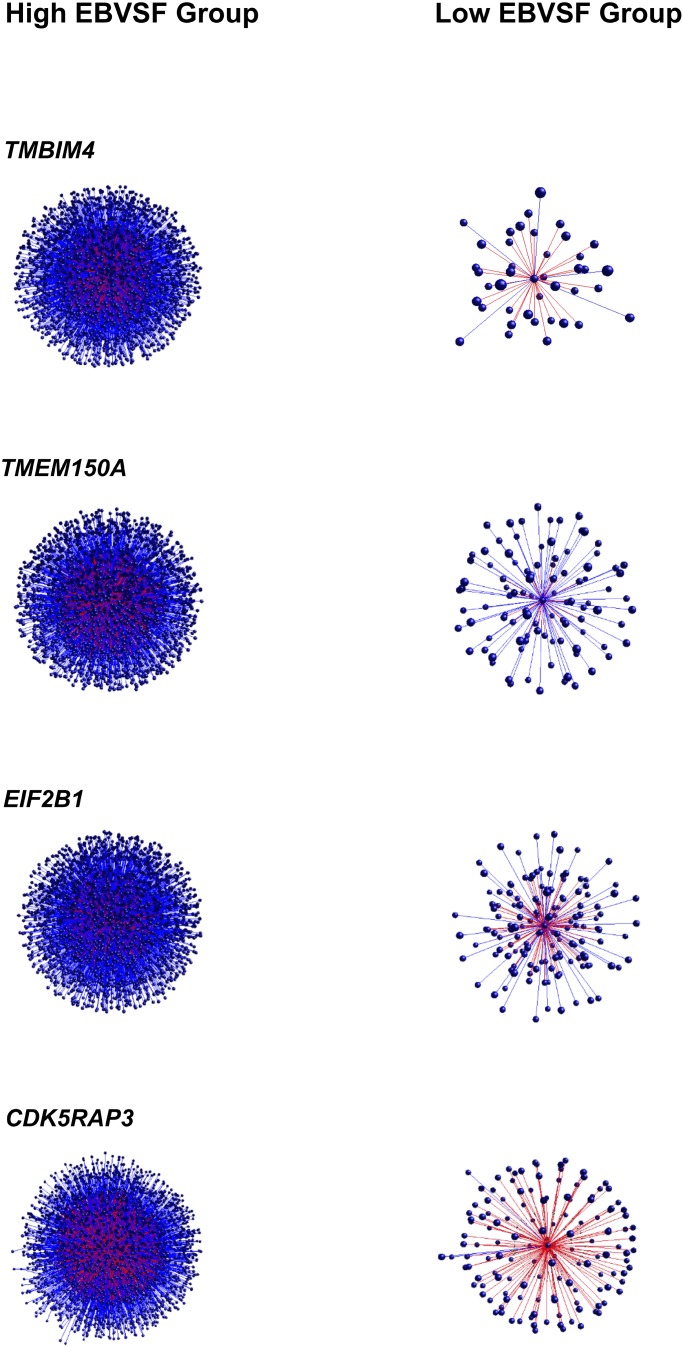
Positive DH between the H and L groups for EBVSF at 14 days of aging. The center spot represents a hub transcript with a high value of DH, the red lines represent positive DH and blue lines represent negative DH. All lines represent significant partial correlations ≥ 0.90 at *p*-value < 0.05 based on the information theory approach, which accounts for all comparisons in the analysis.

Targets of top DH transcripts enriched for terms associated with proteasome GO terms or KEGG pathways. Four transcripts, including: tripartite motif containing 45 (*TRIM45*), ubiquitin specific peptidase 2 (*USP2*), growth factor receptor-bound protein 10 (*GRB10*), and HECT domain-containing e3 ubiquitin protein ligase 4 (*HECTD4*) exhibited negative DH between the High (H) and Low (L) groups for EBVSF at 14 days of aging and were enriched for proteasome (**Figure 1**). The transcripts transmembrane BAX inhibitor motif containing 4 (*TMBIM4*), transmembrane protein 150a (*TMEM150A*), eukaryotic translation initiation factor 2b, subunit 1 alpha, 26kda (*EIF2B1*), and CDK5 regulatory subunit associated protein 3 (*CDK5RAP3*) exhibited positive DH between the high (H) and Low (L) groups for EBVSF at 14 days of aging, and were enriched for terms related to proteasome function (**Figure 2**). **Figures 1**, **2** were constructed using the BioLayout software to visualize DH between the H and L groups for EBVSF at 14 days of aging. The figures selected represented the top DH transcripts related to proteasome function and had the most different networks.

### Potential Regulatory Transcripts Identified by RIF and PIF

Co-expression analyses were performed to identify transcripts associated with differences in the correlation wiring of a network (RIF1) and the magnitude of changes in expression of transcripts associated with tenderness (RIF2). The transcripts identified by RIF1 and RIF2 analyses are shown in **Tables 4**, **5**. A total of 13,485 transcripts identified by RIF1 had negative values, and 52 transcripts had positive values. For RIF2, a total of 50 transcripts identified by RIF2 had negative values, and 13,487 transcripts had positive values.

**Table 4:** Top negative and positive transcripts identified by Regulatory Impact Factor 1 (RIF1) *Z*-scores.

Transcript ID	Gene symbol	Description	RIF1 *Z*-score^∗^
**Top Negative RIF1**			
ENSBTAT00000056983	*HIST1H2AC*	Histone cluster 1, H2ac	−0.038
ENSBTAT00000004571	*SNCG*	Synuclein, gamma (breast cancer-specific protein 1)	−0.038
CUFF.45316	*ABHD8*	Abhydrolase domain containing 8	−0.038
ENSBTAT00000010101	*MKX*	Mohawk homeobox	−0.038
CUFF.13100	*EIF2C2*	Eukaryotic translation initiation factor 2C	−0.038
**Top Positive RIF1**			
ENSBTAT00000012115	*RPS15*	40S ribosomal protein S15	28.779
ENSBTAT00000042285	*bta-mir-133a-2*	bta-mir-133a-2	28.771
CUFF.21214	*MT-CO1*	Mitochondrially encoded cytochrome C oxidase I	28.339
ENSBTAT00000060288	*U6*	U6 spliceosomal RNA	28.182
CUFF.31054	*UBE2D1*	Ubiquitin-conjugating enzyme E2D 1	27.449

*^∗^RIF1 scores are presented as Z score normalized values.*

Functional enrichment analysis was performed by DAVID using the list of the top 3,000 negative and positive for both RIF1 and RIF2. The significant (FDR adjusted *P*-value < 0.05) enriched GO terms are presented in the **Supplementary Table W**. There were no significant enriched GO terms for transcripts with positive RIF1values and negative RIF2 values. Enriched categories for negative RIF1 include the GO BP terms carboxylic acid metabolic process, cellular ketone metabolic process, and lipid metabolic process, while the enriched categories for positive RIF2 include the GO BP terms cellular metabolic process, intracellular organelle, and membrane-bounded organelle (**Supplementary Table W**).

PIF analysis identified 995 significant transcripts (FDR adjusted *P*-value < 0.05) that could impact variation in tenderness. Among the top 10 transcripts identified by PIF, potential biomarkers of tenderness variation included *MB*, *ENO3* and *CA3* (**Table 6**). Functional analysis of significant PIF transcripts identified GO terms involved in the cellular metabolic process, nitrogen compound metabolic process, biosynthetic process and primary metabolic process (FDR adjusted *P*-value < 0.1), and are presented in **Supplementary Table X**.

Some potential regulators identified by both RIF and PIF analyses showed association with mitochondria activity, such as the positive RIF1 transcript mitochondrially encoded cytochrome C oxidase I (*MT-CO1*) (**Table 4**), the positive RIF2 transcripts ATPase family, AAA domain containing 3A (*ATAD3A*) and histidine triad nucleotide binding protein 2 (*HINT2*) (**Table 5**), and two transcripts from cytochromes c oxidase complex identified by PIF: cytochrome C oxidase subunit VIIa polypedtide 1 (muscle) (*COX71A*) and cytochrome C oxidase subunit VIIb (*COX7B*) (**Table 6**).

**Table 5:** Top negative and positive transcripts identified by Regulatory Impact Factor 2 (RIF2) *Z*-scores^∗^.

Transcript ID	Gene symbol	Description	RIF2 Z-score^∗^
**Top Negative RIF2**			
ENSBTAT00000012115	*RPS15*	40S ribosomal protein S15	−16.949
ENSBTAT00000042285	*bta-mir-133a-2*	bta-mir-133a-2	−16.944
CUFF.17073	*LOC618123*	Zinc finger FYVE domain-containing protein 1-like	−16.920
ENSBTAT00000042309	*bta-mir-22*	bta-mir-22	−16.899
CUFF.4368	*SIMC1*	SUMO-interacting motifs containing 1	−16.897
**Top Positive RIF2**			
ENSBTAT00000012676	*ATAD3A*	ATPase family, AAA domain containing 3A	0.151
ENSBTAT00000015208	*HINT2*	Histidine triad nucleotide binding protein 2	0.147
CUFF.45514	*MED26*	Mediator complex subunit 26	0.145
ENSBTAT00000045242	*IFITM1*	Interferon induced transmembrane protein 1	0.061
CUFF.31983	*TAF5L*	TAF5-like RNA polymerase II, p300/CBP-associated factor (PCAF)	0.060

*^∗^RIF2 scores are presented as Z score normalized values.*

**Table 6:** Top 10 transcripts identified by Phenotypic Impact factor (PIF) analysis with FDR adjusted *P*-value < 0.05.

Transcript ID	Gene symbol	Description	PIF	FDR *P*-value^1^
ENSBTAT00000007014	*MB*	Myoglobin	772,384	0.048
ENSBTAT00000007278	*ENO3*	Enolase 3 (beta, muscle)	323,208	0.041
ENSBTAT00000020243	*CA3*	Carbonic anhydrase III, muscle specific	48,453	0.039
ENSBTAT00000026756	*MYBPC2*	Myosin binding protein C, fast type	47,278	0.049
ENSBTAT00000002666	*RPL19*	60S ribosomal protein L19	12,857	0.046
ENSBTAT00000019808	*COX71A*	Cytochrome c oxidase subunit VIIa polypedtide 1 (muscle)	9,309	0.027
CUFF.29715	*HS3ST6*	Heparan sulfate (glucosamine) 3-O-sulfotransferase 6	5,937	0.048
CUFF.8544	*MAP4K4*	Mitogen-activated protein kinase 4	5,880	0.047
ENSBTAT00000053293	*COX7B*	Cytochrome c oxidase subunit VIIb	5,399	0.034
ENSBTAT00000017030	*PDLIM5*	PDZ and LIM domain 5	4,736	0.036

*^1^False Discovery rate adjusted P-value.*

### Transcripts and Pathways Regulated by miRNAs

The miRNAs identified in this study had sequences higher than 65bp, and for this reason they were considered miRNA precursors. *Bta-mir-22* (ENSBTAT00000042309; miRBase; Acc: MI0011372) had 95 bp of transcript length (including UTRs and CDS), and *bta-mir-133a-2* (ENSBTAT00000042285; miRBase; Acc: MI0009732) contained 87 bp of transcript length (including UTRs and CDS) (**Tables 4**, **5**). Since both *bta-mir-133a-2* and *bta-mir-22* were identified as potential regulators by RIF analysis, the target transcripts of this miRNA were identified and analyzed for enriched pathways. The target transcripts of miRNAs *bta-mir-133a-2* and *bta-mir-22* were enriched for 251 and 308 significant IPA pathways, respectively. The complete list of significant IPA pathways is presented in the **Supplementary Tables Y**, **Z**. Both miRNAs identified by the co-expression analysis share the same targets with lethal (*let-7*) miRNA, and the *bta-mir-22* is also a target of *bta-mir-133a-2*. The two miRNAs targeted a variety of structural protein transcripts. The potential regulator *bta-mir-133a-2*, identified by both positive RIF1 and negative RIF2 as a candidate regulator, had 23 myosin transcript targets, including 10 from the myosin heavy chain class, two from the myosin light chain class, and 11 unconventional myosins. The canonical pathway actin cytoskeleton signaling was enriched for both *bta-mir-133a-2* and *bta-mir-22* (*P*-value = 4.90E-06 and 7.24E-08, respectively). The *bta-mir-133a-2* and *bta-mir-22* had some overlapping targets, such as the eukaryotic translation elongation factor 1 alpha 2 (*EEF1A2*), which is a paralog of the eukaryotic translation elongation factor 1 alpha 1 (*EEF1A*), identified as a DE transcript. Another DE transcript targeted by bta-mir-133a-2 was the apolipoprotein L3 (*APOL3*). DH transcript ubiquitin specific peptidase 2 (*USP2*) was a target of *bta-mir-22*, while tripartite motif containing 45 (*TRIM45*) was a transcript target of *bta-mir-133a-2*. Syntaxin-binding protein 6 (amisyn) (*STXBP6*), alpha-kinase 3 (*ALPK3*), and anoctamin 1, calcium activated chloride channel (*ANO1*) were also transcript targets of both miRNAs.

Some of the predicted transcript targets identified by differential expression and co-expression analysis, such as the DE transcript synaptotagmin IV (*SYT4*) and many DH transcripts, were related to calcium signaling. Calpastatin (*CAST*) and two genes from the Calpain family were targets of bta-mir-22, including: calpain 1, (mu/I) large subunit (*CAPN1*) and calpain 11 (*CAPN11*). Two other genes from the Calpain family were transcript targets of both miRNAs: calpain 3, (p94) (*CAPN3*) and the calpain 7 (*CAPN7*). The canonical calcium-signaling pathway was enriched for transcript targets of both *bta-mir-133a-2* (*P*-value = 8.71E-07) and *bta-mir-22* (*P*-value = 4.79E-04). The bta-mir-133a-2 targets also enriched for calcium-induced T lymphocyte apoptosis (*P*-value = 7.76E-04) and calcium transport I (*P*-value = 5.62E-03) pathways.

Multiple proteins related to mitochondrial function were identified by co-expression analyses. The *bta-mir-133a-2* and *bta-mir-22* targets enriched for the mitochondrial L-carnitine shuttle pathway (*P*-value = 3.16E-02 and 1.12E-02, respectively). In addition, while glutathione reductase (*GSR*) was a target of bta-mir-22, glutathione synthetase (*GSS*) was a target of *bta-mir-133a-2*. *Bta-mir-133a-2* had glutathione biosynthesis (*P*-value = 6.03E-02) as a significant enriched pathway. Both miRNAs had glutathione S-transferase mu 4 (*GSTM4*) and glutathione S-transferase, alpha 4 (*GSTA4*) as common targets.

## Discussion

### The Relationship of Differentially Expressed Transcripts With Beef Tenderness

This study integrates DE genes and co-expression analysis associated with SF in Nellore cattle. It also contrasts differences in EBVs to focus on changes in gene expression associated with additive genetic differences in SF phenotypic value. Genetic variation in complex traits can be divided into many components like additive, dominance, and interaction effects of genes (Hill et al., 2008). Complex models fit to identify additive variation are capable of detecting effects that are not exclusively additive, such as epistatic effects, when estimating EBVs. Increasing knowledge about biological pathways and gene networks implies that gene-gene interactions (i.e., epistasis) might be important in trait variation (Hill et al., 2008). Nowadays, there are methods for integrating the “omics” and genetic information, creating systems genetics approaches as potential tools for use in animal breeding to study complex traits (Kadarmideen et al., 2006). The aim of these methodologies is to complement, add, and integrate quantitative genetics and transcriptomics to improve model accuracy, as the RNA-Seq provides an unprecedented level of accuracy and precision for measurements of gene expression profiles (Kadarmideen et al., 2006). In a study of boar taint in non-castrated pigs, the authors used summarized EBV to select animals for transcriptomics analysis (Drag et al., 2017). The co-expression analysis revealed modules that were associated with the summarized EBVs, indicating that EBVs were reliable indicators of phenotype variation (Drag et al., 2017). In addition, other studies successfully linked differences in additive genetic merit (EBV) with RNA-Seq based DE genes, pathways, and network analyses (Kogelman et al., 2014). Thus, these reports corroborate that this approach is reliable for differential expression studies.

The identification of 22 DE genes by QuasiSeq is due to the more conservative approach of this methodology, which adjusts for over-dispersion and provides the additional advantage of sharing information across genes when estimating dispersions (Lund et al., 2012). In a study comparing methodologies for RNA-Seq analysis, QuasiSeq was the top ranked method (Burden et al., 2014). Beef tenderness is a complex trait that can be influenced by many factors (i.e., breed, muscle, nutrition) (Bongiorni et al., 2016). As such, it is not unexpected that the number of DE genes identified as a function of beef tenderness would be prone to large variability based on the influence of these many contributing factors. An RNA-Seq study of beef tenderness was performed between two different Italian breeds, Chianina and Maremmana, and within each breed using LD muscle (Bongiorni et al., 2016). These two breeds were selected for different reasons: Maremmana cattle represent robustness and Chianina cattle represent conformation and fast growth. Regardless of breed, the group tender versus tough showed 494 DE genes (Bongiorni et al., 2016). Within Chianina breed, 607 genes were DE, while 163 genes were identified within the Maremmana breed, showing that the number of DE genes for tenderness can vary in different breeds. Most of the DE genes that differed by breed were involved in the regulation of cellular and biological processes (Bongiorni et al., 2016). Another transcriptomic study of LD muscle in Angus steers using microarray and RT-PCR analysis identified 53 DE genes for beef tenderness (Zhao et al., 2012b). Many of the genes identified in this study were involved in skeletal muscle contraction and lipid metabolism (Zhao et al., 2012b). Finally, an RNA-Seq study of tenderness in Nellore cattle identified 40 DE genes, however, just 17 had known functions (Fonseca et al., 2017). This number of DE genes is consistent with our results, and some of the genes with known functions were also related to ubiquitin metabolism and transport of calcium (Fonseca et al., 2017). A GWAS analysis was previously performed in the same population of Nellore cattle, and many QTLs were identified for meat quality traits. However, just a few QTLs were associated with tenderness, and most with small effects (Tizioto et al., 2013b). A proteomic study was also performed in the same population evaluating *Longissimus thoracis* muscle to identify DE proteins related to meat tenderness in Nellore cattle and only 17 DE spots were identified (Carvalho et al., 2014). These proteins were involved in muscle structure, cell organization, and metabolism (Carvalho et al., 2014). The functional roles of many of the transcripts identified in our study are consistent with the biological roles of genes and proteins previously associated with tenderness. DE transcripts identified included the following: components of the ribosome, serine proteases, and regulators of calcium and lipid transport (Gonçalves, 2015).

Postmortem processes result in changes in structural protein genes, such as shortening of the muscle due to rigor mortis, which in turn influences beef tenderness (Lonergan et al., 2010). In this study, protein synthesis may be a factor impacting muscle SF because of the presence of five ribosomal proteins identified as DE transcripts: 60S ribosomal protein L9 (*RPL9*), 60S ribosomal protein L31 (*RPL31*), 60S ribosomal protein L36 (*RPL36*), 40S ribosomal protein S14 (*RPS14*), and 40S ribosomal protein S15 (*RPS15*) (**Table 2**). These five ribosomal component transcripts plus *EEF1A1* are expressed at higher levels in the L group, which represents the group with more tender meat (lower values of EBVSF at 14 days of aging) (**Supplementary Table B**). Ribosomal proteins interact with elongation factor EEF1 during protein biosynthesis (Graifer and Karpova, 2015). A tenderness study in male and female Qinchuan cattle identified high expression of genes related to translational elongation and protein biosynthesis, such as *EEF1A1*, ribosomal protein S27a (*RPS27A*), and eukaryotic translation initiation factor 1 (*EIF1*) in tender meat (Zhang et al., 2011). Protein synthesis was not measured in our study; however, based on the weight gain observed during the feedlot stage, there is no indication of difference in protein accretion between the L and H groups. Thus, a better hypothesis might be that animals in the L group have a higher rate of protein turnover, which is an essential process for cell survival (Voet et al., 2013).

Serine, 2 (trypsin 2) (*PRSS2*) protein is a serine protease that is involved in many biological processes like collagen metabolic process, proteolysis, serine-type endopeptidase activity, apoptosis, and calcium ion binding (Mihalyi, 1953; Sentandreu et al., 2002; De Bruin et al., 2003; Vandenabeele et al., 2005; Abo-Ismail et al., 2013). Serine proteases are proteolytic enzymes responsible for digestion of polypeptides (Di Cera, 2009). Proteolytic enzymes like calpains are important for the meat aging process. Since calpains are known to be important in meat tenderness, it is plausible that changes in the expression or function of *PRSS2* may impact SF in Nellore cattle (Calkins and Sullivan, 2007). However, although some authors found that serine proteases could make meat more tender and may participate in muscle proteolysis, in this study, *PRSS2* had higher expression in the H group, which represents the group with tougher meat (higher values of EBVSF at 14 days of aging) (Uytterhaegen et al., 1994; Alarcon-Rojo and Dransfield, 1995; Boudida et al., 2016). A previous study performed in the same population identified a quantitative trait loci (QTL) associated with SF at 24 h after slaughter, and the associated SNP within this QTL is located near the serine proteinase inhibitor 2 (*SERPIN2*) on BTA2 (Tizioto et al., 2013b). All LD muscle samples used in this study were collected immediately after slaughter and frozen immediately for posterior RNA extraction, which suggests that at the time of the sample collection, *PRSS2* transcript levels were highly abundant. One possible hypothesis is that *PRSS*2 might have been degraded by the presence of serpins before the aging process starts, making the meat tough. Thus, understanding the high expression level of *PRSS*2 in the H group might be important to determine the if this mechanism impacts beef tenderness differences between *Bos taurus* and *Bos indicus* cattle.

The family of synaptotagmins are transmembrane proteins that act as calcium regulators of exocytosis (Südhof, 2002; Johnson et al., 2010). *SYT4* is a paralog of synaptotagmin 11 (*SYT11*), which is a known as a calcium sensor and plays a role in the regulation of the synaptic transmission (Glavan et al., 2009). Calcium is required to recover from injury, and synaptic exocytosis is a similar process as that used by muscle cells repair the membrane damage (Steinhardt et al., 1994; Lek et al., 2013). Calpains are calcium-binding proteins that can also participate in cell membrane repair (Mellgren et al., 2009; Lek et al., 2013). Calpains and synaptotagmins appear to interact and possibly function in tandem in some cell types (Fukuda and Itoh, 2004; Aganna et al., 2006). As the calpains, synaptotagmins may also be involved in meat tenderness. A SNP in the synaptotagmin 9 (*SYT9*) was described as the most significant SNP associated with slice SF in swine (Nonneman et al., 2013). In our study, *SYT4* had higher expression in the H group, which may indicate that calcium concentration is not significantly reduced in the muscle before the aging process starts. Therefore, the consequent toughness of the meat might be related to understanding why the calcium is not being used by proteolytic enzymes, which is a common process for meat tenderization during the 14 days of aging.

Apolipoproteins are related to lipid transporter activity, and share characteristics with members of the apoptotic Bcl-2 family (Uzureau et al., 2016). Some members of this family like the apolipoprotein A5 (*APOA5*) and apolipoprotein C3 (*APOC3*) are regulators of triglyceride, lipoprotein metabolism, cholesterol, LDL, and HDL (O’Brien et al., 2005). Apolipoproteins were associated with meat tenderness in swine (Hui et al., 2013). SNPs in both *APOA5* and *APOC3* genes were associated with various meat quality traits like cooked weight percentage, drip loss rate, meat color value of LD muscle, and SF (Hui et al., 2013). In this study, the *APOL3* transcript had higher expression in the H groups that may suggest that lipid transportation and metabolism can impact beef tenderness process.

The ATP-binding cassette, sub-family C (CFTR/MRP), member 4 (*ABCC4*), is also known as multidrug resistance-associated protein, which are ATP-dependent-export and anion transporters (Hammond et al., 2007). A substrate of *ABCC4* is glutathione (*GSH*), which exhibits greater excretion in the presence of *ABCC4* (Bai et al., 2004). The export of *GSH* by *ABCC4* is observed in the cell during apoptosis. A decrease of *GSH* can lead to an increase in the presence of reactive oxygen species (ROS) or accelerate the mitochondrial damage. When *GSH* is not exported, cell survival increases (Hammond et al., 2007). In this study, *ABCC4* had higher expression in the L group, indicating a potential increase in the exportation of *GSH* from the cell and higher levels of oxidation. In agreement with this hypothesis, another study in Chianina cattle indicated that the presence of ROS can induce protein fragmentation and consequently impact beef tenderness (D’Alessandro et al., 2012). Oxidative stress was also impacts glycolysis and heat shock proteins responses, which are mechanisms known to positively effect beef tenderness (D’Alessandro et al., 2012). In addition, a previous study performed in the same population identified a QTL that contained the glutathione S-transferase alpha gene family located on BTA23 at 24 Mb that accounted for 0.11% of the additive genetic variance for SF at 24 h of aging (Tizioto et al., 2013b).

Our study indicates that despite the low number of DE genes detected, the DE transcripts identified might trigger molecular mechanisms elucidating the differences in SF between the L and H group in Nellore cattle. The identification of molecular targets can effectively be applied in studies of animal breeding and genetics to understand the differences in the meat quality of Nellore cattle and other breeds.

### Differential Hubbing Transcripts Affecting Beef Tenderness

The DH analysis identified transcripts related to proteasome and ubiquitin function, as well as calcium signaling, consistent with previous associations with SF (Zhao et al., 2012b). Fourteen of the top hub genes identified by PCIT were all associated with protein degradation, indicating a large re-wiring (i.e., change in the correlation) of the protein degradation pathways between the H and L groups. One of these transcripts is *USP2*, which is from the family of de-ubiquitinating enzymes and can be expressed as two distinct isoforms with opposing effects in muscle differentiation. While overexpression of *USP2a* stimulates differentiation, the overexpression of *USP2b* inhibits this process (Wing, 2013). *USP2* was previously associated with beef tenderness in a transcriptome study between male and female Qinchuan cattle (Zhang et al., 2011). In addition, another gene from the same family, ubiquitin specific peptidase 32 (*USP32*), was associated with tenderness in Nellore cattle (Fonseca et al., 2017). *TRIM45*, from the TRIM family ubiquitin ligases, is a component of the ubiquitin-proteasome and is involved in a variety of functions like growth regulation, apoptosis, and may suppress transcriptional activities of mitogen-activated protein kinase (MAPK) signaling pathway targets (Shibata et al., 2012). In agreement with these results, *TRIM45* and another gene from the same family, tripartite motif 32 (*TRIM32*), were respectively associated with beef tenderness in Chianina and Maremmana cattle (Bongiorni et al., 2016).

HECT domain containing E3 ubiquitin protein ligase 4 (*HECTD4*) is an E3 ubiquitin-protein, which was identified as DH. Negative correlates of *HECTD4* enriched for proteasome function using the KEGG pathway database. The ubiquitin-proteasome pathway (UPP) controls the protein turnover and degradation of intracellular proteins (Glickman and Ciechanover, 2002; Lecker et al., 2006). The E3 ubiquitin-protein ligase is a component of the UPP and the main enzyme responsible for the process of degradation (Glickman and Ciechanover, 2002; Lecker et al., 2006). Eukaryotic translation initiation factor 2B, subunit 1 alpha (*EIF2B1*) encodes one of five subunits of eukaryotic initiation factor 2B (eIF2B), which is known to affect global rates of protein synthesis. If protein synthesis is impaired, then eIF2B expression also is reduced as part of the ubiquitin-proteasome system based on studies in rat muscle (Tuckow et al., 2011). Mex-3 RNA binding family member C (*MEX3C*) is also an E3 ubiquitin-protein ligase that plays a role in ubiquitination of proteins, immune response, growth, and adiposity (Jiao et al., 2012a,b; Han et al., 2014; Kuniyoshi et al., 2014). In addition, many other DH transcripts were enriched for ubiquitin-proteasome components or functions (**Supplementary Tables C–V**). Growth factor receptor-bound protein 10 (*GBR10*) is an intracellular adaptor protein that may act as a growth suppressor, and its deletion causes hypermuscularity in mice (Holt et al., 2012). In KEGG pathways, the negatively correlated transcript targets of *GBR10* enriched for proteasome and pyrimidine metabolism, while positively correlated transcripts enriched for endocytosis and ubiquitin-mediated proteolysis. CDK5 regulatory subunit associated protein 3 (*CDK5RAP3*) plays a role in cell signaling pathways, including transcriptional regulation and DNA damage response. *CDK5RAP3* might be more or less susceptible to the ubiquitin proteasome system-mediated protein degradation depending on its interaction with other proteins and regulators (Wu et al., 2010). The positively correlated transcript targets of *CDK5RAP3* enriched for the proteasome pathway, containing ATP-dependent degradation of ubiquitinated, 20S proteasome core, and 19S regulatory complex (RC) components, which are required for ubiquitin-dependent proteolysis and peptide cleavage (Glickman and Ciechanover, 2002) (**Supplementary Tables C–V**).

Besides *TRIM45*, *USP2*, *RTP4*, *GRB10*, *HECTD4*, *EIF2B1, MEX3C*, and *CDK5RAP3* whose targets were enriched for functions related to the proteasome, other target transcripts correlated with other DH transcripts were identified, including: *ALPK3*, transmembrane BAX inhibitor motif containing 4 (*TMBIM4*), receptor (chemosensory) transporter protein 4 (*RTP4*), syntaxin binding protein 6 (amisyn) (*STXBP6*), transmembrane protein 150a (*TMEM150A*), ectonucleotide pyrophosphatase/phosphodiesterase 4 (putative) (*ENPP4*), and solute carrier family 25, member 44 (*SLC25A44*) (**Supplementary Tables C–V**). The eight hub genes presented in **Figures 1**, **2** are protein-coding genes according to Biomart Ensembl annotation, and most of them are linked to proteasome in the literature, except for two (*TMBIM4* and *TMEM150A*). Many of the DH transcripts correlated with the proteasome KEGG pathway were not previously identified as associated with beef tenderness and thus might be involved in the protein degradation process of tenderization.

Two hub genes were related to calcium-activated mechanisms: *ANO1* and *TMBIM4*. *ANO1* is a calcium-activated chloride channel, which is essential for numerous physiological functions (Caputo et al., 2008). This gene could be a potential regulator of beef tenderness as the infusion of calcium chloride in meat increases tenderness (Koohmaraie, 1994). Calcium chloride injections in muscle increased the degradation of filamin (a large actin-binding protein), stimulating proteolysis and tenderization (Uytterhaegen et al., 1994; Harris et al., 2001; Lonergan et al., 2010). *TMBIM4* is an anti-apoptotic protein that modulates calcium entry, promotes calcium release from intracellular reserves, and plays a role in cell survival (Gubser et al., 2007). *TMBIM4* is expressed in several tissues including skeletal muscle in humans and also encodes a protein that contains transmembrane domains (Lisak et al., 2015). This gene is known as Golgi anti-apoptotic proteins (GAAP) in humans and may activate calpain 2, promote cell adhesion, and migration by controlling calcium concentration (Saraiva et al., 2013).

Differential hubbing analysis indicated that many potential regulators of beef tenderness are involved in the protein degradation process, and a few DH transcripts are related to calcium-activated mechanisms. Most of these transcripts were not identified in previous studies of SF, and offer novel insights into future research on SF and meat quality.

### Co-expression Analysis Indicates microRNAs Impact Beef Tenderness

In muscle, miRNAs have been suggested to play a role in control gene expression impacting variation in beef tenderness (Zhao et al., 2012a). Several intriguing candidate transcripts were identified as possible regulators of variation in SF in this study, including *bta-mir-133a-2* and *bta-mir-22*. The methodology used for library preparation and sequencing was a standard protocol for mRNA analysis. Although this study was not designated to find miRNAs, the procedure allowed the identification of two miRNAs precursors. Most miRNAs are transcribed from long primary miRNA (pri-miRNA) transcripts, which contains the mature miRNA as part of a predicted RNA hairpin (Zeng et al., 2005). The pri-miRNAs are then cleaved into ∼70 nt hairpin precursor miRNAs that might be able to rescue the miRNA function (Zeng et al., 2005; Van Wynsberghe et al., 2011). In agreement with this finding, other authors have also identified miRNAs using standard library preparation and sequencing for RNA-Seq analysis (Armenta-Medina et al., 2017; Lepe-Soltero et al., 2017). In addition, one miRNA was identified in the PIF analysis in a study of gene co-expression analysis, using the same RNA-Seq protocol, showing that it is possible to identify miRNAs or miRNA precursors even if the experiment is not designed to sequence miRNAs (Cesar et al., 2015). The *bta-mir-133a-2* and *bta-mir-22* were only identified in to the RIF analysis, and were among the top 10 potential regulators (based on extreme RIF *Z* score). The *bta-mir-133a-2* was identified as both an extreme positive RIF1 and extreme negative RIF2 candidate regulatory gene. For this reason, these two miRNAs might be crucial regulators, as they have many target genes and pathways that can be involved in the control of meat tenderness. Functional analysis of miRNAs targets indicated that pathways related to calcium signaling, proteolysis, and apoptosis are impacted by fluctuations in *bta-mir-133a-2* and *bta-mir-22*. In addition, both RIF and PIF co-expression analysis corroborate with the DH transcripts identified, indicating that protein degradation and calcium activity are key mechanisms to understand the differences between the H and L SF groups.

RIF1 results identified ubiquitin-conjugating enzyme E2D1 (*UBE2D1*). This gene promotes the ubiquitination of the 26S proteasome in cells (Jacobson et al., 2014). *UBE2D1* may regulate SF variability since this gene encodes for a protein involved in proteasome function. Other biologically interesting RIF1 candidates identified included: histone cluster 1, H2ac (*HIST1H2AC)*; synuclein, gamma (breast cancer-specific protein 1) (*SNCG)*; and mohawk homeobox (*MKX*). Histones are proteins that play a role in transcriptional regulation, chromatin organization, and DNA packaging (Keogh et al., 2017). Genes coding for histone proteins like *HIST1H2AC* were up-regulated in skeletal muscle tissue of cattle expressing compensatory growth in Holstein Friesian bulls, suggesting an increase in feed efficiency in cattle (Keogh et al., 2017). *SNCG* increases metastasis at the cellular level and regulates many pathways including cell motility (Miao et al., 2014). *MKX* is a potential transcriptional repressor and critical regulator of gene expression that can repress myogenic differentiation (*MYOD*) gene expression in mice and negatively regulate muscle differentiation (Anderson et al., 2012; Chuang et al., 2014). Reduced expression of *MYOD* in mice muscle might be associated with tenderness (Lian et al., 2013). Interesting RIF2 candidates identified included: ATPase family, AAA domain containing 3A (*ATAD3A*); interferon induced transmembrane protein 1 (*IFITM1)*; and TAF5-like RNA polymerase II, p300/CBP-associated factor (PCAF)-associated factor, 65kDa (*TAF5L)*. *ATAD3* is a member of the ATPases family, a highly conserved ubiquitous protein associated with development and mitochondrial DNA integrity (Issop et al., 2015). *IFITM1* impacts cell growth. A transcriptomic analysis combined with RIF was performed in Blond d’Aquitaine and Charolais during pre-natal development and identified interferon signaling molecules as upregulated in the Blond d’Aquitaine breed compared to Charolais (Cassar-Malek et al., 2017). Interferon signaling has been suggested to play a role in the formation of new muscle fibers (Cassar-Malek et al., 2017). *TAF5L* is involved in the regulation of myogenic transcription, and *MYOD* function is enhanced by PCAF and the transcriptional coactivators p300 (Kuninger et al., 2006). Thus, it is plausible that these genes may impact muscle growth and subsequently tenderness.

In other species, *miR-133a* can promote muscle regeneration in skeletal muscle and regulates cardiac and muscle remodeling (Roderburg et al., 2013). The *miR-133a* is associated with fibrosis in liver and its overexpression can decrease the expression of collagens, which might indicate similar function in muscle (Roderburg et al., 2013). Both fibrosis and collagen expression could impact muscle tenderness. *MiR-133a* is a muscle-specific miRNA, or myomiR, which plays an important role in skeletal muscle proliferation and regeneration (Horak et al., 2016). Among the roles of myomiRs, *miR-133a-2* promotes myoblast proliferation, regulates alternative splicing, is pro-apoptotic, controls mitochondrial metabolism, and impacts muscle fiber type (Horak et al., 2016). In addition, *miR-133* is negatively regulated by mechanistic target of rapamycin (serine/threonine kinase) (MTOR), which regulates apoptosis, plays a role in myogenesis, and might control cell proliferation and differentiation (Horak et al., 2016).

In humans, *miR-22*, also a myomiR, impacts cell proliferation and apoptosis (Bersani et al., 2016). The role of *bta-mir-22* has not been well characterized in skeletal muscle, but it plays a role in calcium signaling in the heart (Gurha et al., 2012). Consistent with the known function of *bta-mir-22*, target transcripts identified in this study were involved in calcium signaling. Transcript targets for both *bta-mir-22* and *bta-mir-133a-2* included calcium transporters such as the calcium channel, voltage-dependent, N type, alpha 1B subunit (*CACNA1B*) and ATPase, calcium-transporting, plasma membrane 2 (*ATP2B2*). Transcripts activated by calcium or calcium-binding such as calcium/calmodulin-dependent protein kinase ID (*CAMK1D*), calcium/calmodulin-dependent protein kinase kinase 2, beta (*CAMKK2*), and calcium-dependent secretion activator 2 (*CADPS2*) were targets of both miRNAs. Calpain 5 (*CAPN5*) and calpain 15 (*CAPN15*) were targets of *bta-mir-133a-2*, while calpain 7 (*CAPN7*) was target of *bta-mir-22*. Interestingly, *SYT4* identified in the differential expression analysis was also targeted by *bta-mir-22*.

Apoptosis is known to be an important process for beef tenderness and to be involved in the aging process of meat (Ouali et al., 2006). Both miRNAs had many transcript targets that are anti- or pro-apoptotic, such as Bcl2-like 1 (*BCL2L1*) that inhibits cell death, and Bcl2-like 13 (apoptosis facilitator) (*BCL2L13*), which promotes apoptosis (Kataoka et al., 2001). Other transcript targets identified in the RIF co-expression analysis included programmed cell death 6 interacting protein (*PDCD6IP*) and programmed cell death 10 (*PDCD10*). In agreement with these findings, a protein study of SF in Nellore cattle identified heat shock protein 27 (*HSP27)* and heat shock protein 70 (*HSP70*) as having anti-apoptotic functions (Carvalho et al., 2014). However, the two proteins had differing effects. While *HSP27* may contribute to increased tenderness, *HSP70* is suggested to negatively impact tenderness (Carvalho et al., 2014).

Among the top 10 PIF transcripts, myoglobin (*MB*) had the highest ranking, which implicates it as the most important transcript related to the SF variation based on this method. *MB* is a cytoplasmic hemoprotein, which can serve as an oxygen reservoir releasing oxygen during periods of hypoxia or anoxia (Ordway and Garry, 2004). It can keep the intracellular concentration of oxygen constant even in high levels of activity (Ordway and Garry, 2004). *MB* has a similar role to that of creatine phosphokinase, which increases ATP concentrations during muscle activity; however, *MB* increases the oxygen concentrations instead of ATP (Ordway and Garry, 2004). Oxidative energy metabolism is important to the transformation of muscle into meat. When cells are deprived of oxygen, ATP synthesis occurs via anaerobic conditions with glycolytic phosphorylation by creatine (Ouali et al., 2006). In a study of beef tenderness in Chianina cattle, expression of creatine was higher in the tender group (D’Alessandro et al., 2012). *MB* was also a target of *bta-mir-22*, present in high quantities in type I muscle fibers, and responsible for oxygen storage and transport (Picard et al., 2011). Type I fibers were associated with beef tenderness, as increased type I fibers results in more tender beef (Strydom et al., 2000; Renand et al., 2001; Choi and Kim, 2009). In addition, myoglobin is involved in iron ion binding. The presence of iron may impact beef tenderness as markers in the Cast and mu-calpain (*CAPN4751*) genes were associated with iron levels (Casas et al., 2014). Thus, changes in myoglobin may act as a surrogate to track differences in fiber type between the extreme tenderness samples used in this study.

High glycolytic metabolism and oxidative metabolism are dependent on muscle type and breed (Cassar-Malek and Picard, 2016). For example, enolase 3 (beta, muscle) (*ENO3*) was positively correlated with tenderness in glycolytic muscles, like *semitendinosus*, and in breeds that present glycolytic muscle properties like Limousin (Cassar-Malek and Picard, 2016). In another study, two isoforms of the glycolytic enzyme enolase (*ENO1* and *ENO3*) were associated with tenderness in Charolais (Guillemin et al., 2011). However, *ENO1* and *ENO3* have different biological functions. *ENO1* (alpha, muscle) is a transcriptional regulator involved in the immunoglobulin response, while *ENO3* (beta, muscle) is involved in the development and regeneration of muscle. Also, *ENO3* was more abundant than *ENO1* in steers compared to young bulls (Guillemin et al., 2011). Among the top PIF transcripts, *ENO3* had the second highest score, and carbonic anhydrase III (*CA3*) had the third highest score in the ranking. *CA3* is a high-speed enzyme involved in proton homeostasis and converting carbohydrate into carbon dioxide. Changes in proton homeostasis decrease the electrostatic repulsion of myofibrillar proteins and facilitate the lateral shrinkage of muscle fibers (sarcomere length) (Pearce et al., 2011). The high presence of the glycolytic enzyme *CA3* is also associated with increased meat tenderness (Laville et al., 2009; Choi et al., 2010; D’Alessandro et al., 2012). Two other transcripts from the *CA* family were targets of *bta-mir-22*: carbonic anhydrase VIII (*CA8*) and carbonic anhydrase XII (*CA12*). Results found in the RIF and PIF co-expression analysis identified two miRNAs as potential regulators and also confirmed that some transcripts like *MB*, *ENO3*, and *CA3*, previously described by other studies, are good markers of tenderness.

Proteolysis impacts the structure of the sarcomere/myofibril (Ertbjerg and Puolanne, 2017). The thin filaments are made of the protein actin, while the thick filaments are composed by the protein myosin (Ertbjerg and Puolanne, 2017). PIF analysis identified myosin-binding protein C, fast type (*MYBPC2)* as a candidate biomarker for tenderness. Myosin is an important structural protein of the thick filament of the sarcomere, responsible for contraction velocity and power (Zhao et al., 2012b). Both myosin and actin interact for contraction to occur, forming the complex called actomyosin that is ATP dependent, which means that the actin and myosin can dissociate in the presence of ATP (Goll et al., 1984). Actomyosin bonds become irreversible in postmortem muscles when the supply of ATP is depleted. Myosin heavy chains (*MyH3*) are present in the tender meat due to the proteolytic activity of the muscle that continues postmortem (Zhao et al., 2012b). The amount that a myofibril shortens affects tenderness, and meat with very short sarcomeres might be tougher (Locker and Hagyard, 1963). Myosin regulatory light chain (*MLC2*) and myosin binding protein H (*MyBP-H*) expression are also associated with meat tenderness (Carvalho et al., 2014). In addition, histone methylation and modifications are related to beef tenderness in Angus based on a study in fast and slow skeletal muscle fibers, which identified differential histone modification patterns in myosin heavy chain genes (Zhao et al., 2015).

## Conclusion

Differential gene expression analysis identified transcripts that could be responsible for variation in beef tenderness in Nellore cattle, which differ from those previously identified in *Bos taurus* breeds. Systems biology analyses using PCIT, PIF and RIF helped to elucidate differential gene networks that may be associated with beef tenderness, many of which were related to proteolysis, calcium signaling, and apoptosis. Furthermore, two microRNAs, *bta-mir-133a-2* and *bta-mir-22*, were identified in the RIF analysis as potential regulators of tenderness. These miRNA were associated with proteolysis and apoptosis as well as a number other genes identified in this study to impact tenderness. While most of the genes identified in this study have not been previously implicated in beef tenderness, we conclude that the identified pathways comprised by these genes were consistent and interconnected with previously identified genes that regulate beef tenderness. These findings provide a better understanding of the molecular mechanisms involved in the regulation of beef tenderness in Nellore cattle.

## Data Availability Statement

The dataset supporting the conclusions of this article is available in the European Nucleotide Archive (ENA) repository (EMBL-EBI), under accession PRJEB13188, PRJEB10898, and PRJEB19421 (https://www.ebi.ac.uk/ena/submit/sra/).

## Author Contributions

TG performed the bioinformatics and data analysis, and wrote the manuscript. LdAR co-designed the experiment and co-directed the work. JK, AC, and SdSA contributed to the bioinformatics and data analyses. GBM co-designed the experiment. GG contributed to the sequencing analysis. GCM revised all the drafts of the manuscript. EF-W contributed to the data analysis. JR supervised the data analysis. LC supervised the data analysis, co-designed the experiment, and co-directed the work. All the authors have read and approved the final manuscript.

## Conflict of Interest Statement

The authors declare that the research was conducted in the absence of any commercial or financial relationships that could be construed as a potential conflict of interest.

## Abbreviations

DE: differentially expressed
DH: differentially hubbed
EBVSF: shear force estimated breeding values
H: highest values of EBVSF
L: lowest values of EBVSF
LD: *Longissimus dorsi*
PCIT: partial correlation with information theory
PIF: phenotypic impact factor
RIF: regulatory impact factor
SF: shear force.
