# Supplementary material for: Gene Co-expression Analysis Indicates Potential Pathways and Regulators of Beef Tenderness in Nellore Cattle
Source: Front Genet. 2018 Oct 5;9:441. doi: 10.3389/fgene.2018.00441 (PMC6182065; doi:10.3389/fgene.2018.00441)
Supplement: Supplementary file 1 [file Data_Sheet_1.docx]

Supplementary Material

**Gene Co-expression Analysis Indicates Potential Pathways and Regulators of Beef Tenderness in Nellore Cattle**

Tássia Mangetti Gonçalves^1^, Luciana Correia de Almeida Regitano ^2^, James E. Koltes ^3^, Aline Silva Mello Cesar^1^, Sónia Cristina da Silva Andrade^1,4^, Gerson Barreto Mourão^1^, Gustavo Gasparin^1^, Gabriel Costa Monteiro Moreira^1^, Elyn Fritz-Waters^3^, James M. Reecy^3^, Luiz Lehmann Coutinho^1*^

*** Correspondence:** Dr. Luiz Lehmann Coutinho: llcoutinho@usp.br

# Supplementary Data

**Table A.** Summary of total alignment statistics and read counts of the 24 samples with extreme EBVSF values at 14 days of aging.

| Sample^1^ | Total Reads^2^ | Filtered Reads^3^ | Aligned Reads | Aligned Reads (%) | Uniquely Aligned Reads | Uniquely Aligned Reads (%) |
| --- | --- | --- | --- | --- | --- | --- |
| H31 | 23,492,238 | 16,904,322 | 13,544,569 | 80.12 | 11,250,072 | 83.06 |
| H69 | 35,652,960 | 24,877,628 | 19,917,630 | 80.06 | 14,961,594 | 75.12 |
| H83 | 28,410,964 | 18,935,924 | 13,282,443 | 70.14 | 10,969,664 | 82.59 |
| H104 | 32,984,664 | 23,569,408 | 18,878,494 | 80.10 | 16,077,298 | 85.16 |
| H220 | 29,502,198 | 23,582,052 | 22,794,116 | 96.66 | 15,127,692 | 66.37 |
| H249 | 35,961,328 | 23,177,167 | 17,257,464 | 74.46 | 12,527,418 | 72.59 |
| H251 | 36,891,122 | 25,386,891 | 20,034,936 | 78.92 | 15,703,342 | 78.38 |
| H255 | 21,802,954 | 18,475,678 | 16,683,311 | 90.30 | 12,460,588 | 74.69 |
| H286 | 34,270,230 | 23,925,670 | 16,693,167 | 69.77 | 11,668,652 | 69.90 |
| H293 | 32,972,338 | 23,434,347 | 18,148,181 | 77.44 | 13,956,904 | 76.91 |
| H300 | 31,790,744 | 23,029,262 | 18,173,808 | 78.92 | 15,344,620 | 84.43 |
| L68 | 19,501,564 | 16,388,331 | 15,353,150 | 93.68 | 11,917,196 | 77.62 |
| L71 | 31,521,041 | 26,550,166 | 24,559,761 | 92.50 | 17,671,080 | 71.95 |
| L89 | 28,659,904 | 20,240,793 | 15,696,120 | 77.55 | 12.125,914 | 77.25 |
| L92 | 17,829,140 | 14,873,842 | 13,953,405 | 93.81 | 10,648,046 | 76.31 |
| L95 | 27,709,774 | 23,190,819 | 20,997,130 | 90.54 | 16,832,520 | 80.17 |
| L141 | 40,078,620 | 28,321,605 | 21,963,600 | 77.55 | 16,083,708 | 73.23 |
| L145 | 51,339,112 | 41,367,060 | 38,242,577 | 92.45 | 28,571,676 | 74.71 |
| L152 | 43,517,182 | 34,779,693 | 31,877,993 | 91.66 | 22,875,704 | 71.76 |
| L186 | 31,558,052 | 22,020,573 | 17,180,737 | 78.02 | 12,809,414 | 74.56 |
| L208 | 28,136,190 | 22,911,545 | 21,064,220 | 91.94 | 16,557,144 | 78.60 |
| L223 | 21,781,686 | 16,585,135 | 13,335,412 | 80.41 | 11,102,826 | 83.25 |
| L248 | 31,507,614 | 23,182,290 | 18,947,995 | 81.73 | 16,392,278 | 86.51 |
| L268 | 22,059,752 | 17,765,273 | 16,693,167 | 93.97 | 11,668,652 | 69.90 |

^1^Samples that start with L and H have low and high EBVSF values at 14 days of aging, respectively.

^2^Total reads before the Seqyclean filter.

^3^Number of reads after the Seqyclean filter.

**Table B.** Relative expression levels of the differentially expressed transcripts obtained between H and L groups for EBVSF at 14 days of aging in Nellore steers with q-value < 0.10.

| **Gene symbol** | **Transcript ID** | **Counts^1^ H group** | **Counts L group** | **Log2FC**  **(L/H) ^2^** | **q-value^3^** |
| --- | --- | --- | --- | --- | --- |
| *SYT4* | ENSBTAT00000002357 | 50.64 | 39.23 | -0,37 | 7.78E-07 |
| *DCSTAMP* | ENSBTAT00000031443 | 14.64 | 3.08 | -2,25 | 1.16E-06 |
| *PRSS2* | ENSBTAT00000028731 | 30.00 | 17.62 | -0,77 | 5.77E-06 |
| *RPL9* | ENSBTAT00000064379 | 3.18 | 9.85 | 1,63 | 4.82E-05 |
| *RPS15* | ENSBTAT00000012115 | 2.36 | 3.38 | 0,52 | 6.39E-05 |
| *AP1M1-golgi* | CUFF.45691 | 1.36 | 3.00 | 1,14 | 6.43E-05 |
| *RPL36* | ENSBTAT00000050149 | 4.92 | 9.07 | 0,88 | 1.00E-04 |
| *ACOT11* | CUFF.1369 | 0.73 | 4.00 | 2,45 | 2.00E-04 |
| *7SK* | ENSBTAT00000060649 | 1.29 | 3.17 | 1,30 | 5.00E-04 |
| *RPL31* | ENSBTAT00000064020 | 2.09 | 2.77 | 0,41 | 5.00E-04 |
| *RPS14* | ENSBTAT00000027356 | 2.03 | 3.45 | 0,77 | 1.00E-03 |
| *ABCC4* | ENSBTAT00000010182 | 22.12 | 38.12 | 0,79 | 2.60E-03 |
| *PGM2L1* | CUFF.20989 | 1.64 | 4.92 | 1,58 | 3.40E-03 |
| *APOL3* | ENSBTAT00000016190 | 4.73 | 4.69 | -0,01 | 3.80E-03 |
| *SNORD113* | ENSBTAT00000059671 | 2.81 | 1.75 | -0,68 | 3.90E-03 |
| Unannotated^4^ | CUFF.14892 | 4.36 | 6.38 | 0,55 | 4.70E-03 |
| *EEF1A1* | ENSBTAT00000030625 | 0.18 | 5.69 | 4,98 | 2.75E-02 |
| *JAM2* | CUFF.2483 | 2.91 | 3.43 | 0,24 | 6.73E-02 |
| *B4GALNT2* | ENSBTAT00000013010 | 0.52 | 3.98 | 2,94 | 7.27E-02 |
| *MT-ATP6* | CUFF.14879 | 259.80 | 138.44 | -0,91 | 7.41E-02 |
| *EME2* | ENSBTAT00000022021 | 2.93 | 2.84 | -0,05 | 8.18E-02 |
| *KIAA1456* | ENSBTAT00000011782 | 3.75 | 2.47 | -0,60 | 9.73E-02 |

^1^Average of normalized counts for both H and L groups.

^2^Log2FC (L/H) was the log2 Fold Change between L and H groups for EBVSF at 14 days of aging.

^3^q-value methodology was used to control the false discovery rate (FDR) at 10%.

^4^CUFF.14892 was not able to be annotated using Genbank and Ensembl databases.

**Table C.** Top informative DAVID ALL categories including Gene Ontology (GO) biological processes (BP) and molecular function (MF) enriched among all transcripts negatively and positively correlated with Mex-3 RNA binding family member C (*MEX3C*).

| Category | Term | Count | % | P-value | FDR P-value^1^ |
| --- | --- | --- | --- | --- | --- |
| Negatively correlated | | | | | |
| GOTERM_BP_ALL | cellular process | 387 | 36.4 | 4.80E-15 | 9.20E-12 |
| GOTERM_BP_ALL | cellular metabolic process | 270 | 25.4 | 1.80E-14 | 1.70E-11 |
| GOTERM_BP_ALL | metabolic process | 316 | 29.7 | 2.50E-11 | 1.60E-08 |
| GOTERM_BP_ALL | cellular macromolecule metabolic process | 198 | 18.6 | 5.20E-10 | 2.50E-07 |
| GOTERM_BP_ALL | primary metabolic process | 269 | 25.3 | 9.80E-10 | 3.70E-07 |
| GOTERM_MF_ALL | protein binding | 299 | 28.1 | 3.10E-09 | 2.10E-06 |
| GOTERM_BP_ALL | cellular protein metabolic process | 129 | 12.1 | 1.40E-08 | 4.50E-06 |
| GOTERM_BP_ALL | macromolecule metabolic process | 214 | 20.1 | 1.10E-07 | 3.00E-05 |
| GOTERM_BP_ALL | cellular catabolic process | 50 | 4.7 | 3.20E-06 | 7.70E-04 |
| GOTERM_BP_ALL | protein metabolic process | 143 | 13.5 | 8.10E-06 | 1.70E-03 |
| KEGG_PATHWAY | Proteasome | 11 | 1 | 9.80E-05 | 1.60E-02 |
| Positively correlated | | | | | |
| GOTERM_BP_ALL | cellular process | 183 | 26.3 | 1.20E-06 | 1.60E-03 |
| GOTERM_MF_ALL | binding | 258 | 37.1 | 3.50E-05 | 1.60E-02 |
| GOTERM_BP_ALL | cellular macromolecule metabolic process | 94 | 13.5 | 5.30E-05 | 3.40E-02 |
| KEGG_PATHWAY | Insulin signaling pathway | 12 | 1.7 | 6.60E-04 | 4.20E-02 |
| KEGG_PATHWAY | Neurotrophin signaling pathway | 12 | 1.7 | 4.00E-04 | 5.10E-02 |
| GOTERM_BP_ALL | cellular metabolic process | 119 | 17.1 | 1.30E-04 | 5.50E-02 |
| GOTERM_MF_ALL | phosphatase activity | 15 | 2.2 | 1.10E-03 | 9.50E-02 |
| GOTERM_MF_ALL | nucleotide binding | 67 | 9.6 | 1.10E-03 | 8.10E-02 |

^1^False Discovery rate adjusted P-value.

**Table D.** Top informative DAVID ALL categories including Gene Ontology (GO) biological processes (BP) and molecular function (MF) enriched among all transcripts negatively and positively correlated with protein tyrosine phosphatase, non-receptor type 3 (*PTPN3*).

| Category | Term | Count | % | P-value | FDR P-value^1^ |
| --- | --- | --- | --- | --- | --- |
| Negatively correlated | | | | | |
| GOTERM_MF_ALL | binding | 555 | 47.6 | 2.00E-11 | 1.40E-08 |
| GOTERM_MF_ALL | protein binding | 316 | 27.1 | 5.40E-09 | 1.90E-06 |
| GOTERM_BP_ALL | cellular process | 376 | 32.2 | 8.50E-07 | 1.70E-03 |
| GOTERM_BP_ALL | macromolecule localization | 61 | 5.2 | 1.20E-06 | 1.20E-03 |
| GOTERM_BP_ALL | protein transport | 49 | 4.2 | 2.00E-06 | 1.30E-03 |
| GOTERM_BP_ALL | establishment of protein localization | 49 | 4.2 | 2.10E-06 | 1.00E-03 |
| GOTERM_BP_ALL | protein localization | 52 | 4.5 | 3.10E-06 | 1.20E-03 |
| GOTERM_BP_ALL | intracellular transport | 36 | 3.1 | 1.90E-05 | 6.30E-03 |
| GOTERM_BP_ALL | regulation of biological process | 229 | 19.6 | 3.60E-05 | 1.00E-02 |
| GOTERM_BP_ALL | regulation of cellular process | 217 | 18.6 | 4.60E-05 | 1.10E-02 |
| Positively correlated | | | | | |
| GOTERM_BP_ALL | cellular process | 53 | 40.5 | 2.70E-05 | 1.70E-02 |
| GOTERM_BP_ALL | cellular macromolecule metabolic process | 30 | 22.9 | 4.00E-04 | 8.40E-02 |

^1^False Discovery rate adjusted P-value.

**Table E.** Top informative DAVID ALL categories including Gene Ontology (GO) biological processes (BP), molecular function (MF), and KEGG pathways enriched among all transcripts negatively and positively correlated with tripartite motif containing 45 (*TRIM45*).

| Category | Term | Count | % | P-value | FDR P-value^1^ |
| --- | --- | --- | --- | --- | --- |
| Negatively correlated | | | | | |
| GOTERM_BP_ALL | cellular process | 373 | 35 | 2.80E-13 | 5.60E-10 |
| GOTERM_MF_ALL | protein binding | 309 | 29 | 4.90E-13 | 3.30E-10 |
| GOTERM_BP_ALL | cellular metabolic process | 255 | 23.9 | 9.60E-12 | 9.60E-09 |
| GOTERM_BP_ALL | cellular macromolecule metabolic process | 191 | 17.9 | 3.00E-09 | 2.00E-06 |
| GOTERM_BP_ALL | metabolic process | 294 | 27.6 | 1.10E-07 | 5.40E-05 |
| GOTERM_BP_ALL | primary metabolic process | 252 | 23.6 | 3.30E-07 | 1.30E-04 |
| GOTERM_BP_ALL | macromolecule metabolic process | 204 | 19.1 | 1.50E-06 | 5.10E-04 |
| GOTERM_BP_ALL | cellular protein metabolic process | 117 | 11 | 3.80E-06 | 1.10E-03 |
| GOTERM_MF_ALL | binding | 488 | 45.7 | 6.10E-06 | 2.10E-03 |
| GOTERM_BP_ALL | protein localization | 48 | 4.5 | 7.20E-06 | 1.80E-03 |
| KEGG_PATHWAY | Proteasome | 11 | 1 | 6.90E-05 | 1.10E-02 |
| Positively correlated | | | | | |
| GOTERM_MF_ALL | zinc ion binding | 40 | 12.3 | 2.00E-06 | 6.60E-04 |
| GOTERM_BP_ALL | cellular metabolic process | 67 | 20.6 | 9.70E-06 | 8.60E-03 |
| GOTERM_BP_ALL | cellular process | 93 | 28.5 | 1.10E-05 | 5.00E-03 |
| GOTERM_MF_ALL | binding | 129 | 39.6 | 1.20E-05 | 1.90E-03 |
| GOTERM_MF_ALL | transition metal ion binding | 42 | 12.9 | 6.30E-05 | 6.70E-03 |
| GOTERM_BP_ALL | nitrogen compound metabolic process | 35 | 10.7 | 2.60E-04 | 7.30E-02 |
| GOTERM_BP_ALL | cellular nitrogen compound metabolic process | 34 | 10.4 | 2.90E-04 | 6.30E-02 |
| GOTERM_MF_ALL | nucleic acid binding | 44 | 13.5 | 4.10E-04 | 3.30E-02 |
| GOTERM_BP_ALL | regulation of RNA metabolic process | 24 | 7.4 | 4.60E-04 | 7.70E-02 |
| GOTERM_MF_ALL | DNA binding | 28 | 8.6 | 4.80E-04 | 3.10E-02 |

^1^False Discovery rate adjusted P-value.

**Table F.** Top informative DAVID ALL categories including Gene Ontology (GO) biological processes (BP), molecular function (MF), and KEGG pathways enriched among all transcripts negatively and positively correlated with ubiquitin specific peptidase 2 (*USP2*).

| Category | Term | Count | % | P-value | FDR P-value^1^ |
| --- | --- | --- | --- | --- | --- |
| Negatively correlated | | | | | |
| GOTERM_BP_ALL | cellular process | 354 | 37.1 | 1.10E-13 | 2.20E-10 |
| GOTERM_MF_ALL | protein binding | 285 | 29.8 | 1.10E-11 | 7.40E-09 |
| GOTERM_BP_ALL | cellular metabolic process | 231 | 24.2 | 6.80E-09 | 6.60E-06 |
| GOTERM_BP_ALL | cellular macromolecule metabolic process | 177 | 18.5 | 3.80E-08 | 2.50E-05 |
| GOTERM_BP_ALL | macromolecule localization | 55 | 5.8 | 1.20E-06 | 5.70E-04 |
| GOTERM_BP_ALL | protein localization | 48 | 5 | 1.30E-06 | 4.90E-04 |
| GOTERM_BP_ALL | protein transport | 44 | 4.6 | 2.70E-06 | 8.70E-04 |
| GOTERM_BP_ALL | establishment of protein localization | 44 | 4.6 | 2.80E-06 | 8.00E-04 |
| GOTERM_BP_ALL | cellular process | 191 | 20 | 4.30E-06 | 9.50E-04 |
| GOTERM_BP_ALL | primary metabolic process | 233 | 24.4 | 4.20E-06 | 1.00E-03 |
| KEGG_PATHWAY | Proteasome | 11 | 1.2 | 2.30E-05 | 3.60E-03 |
| Positively correlated | | | | | |
| GOTERM_BP_ALL | cellular metabolic process | 67 | 22 | 3.10E-06 | 3.00E-03 |
| GOTERM_BP_ALL | primary metabolic process | 67 | 22 | 6.30E-05 | 3.00E-02 |
| GOTERM_BP_ALL | cellular macromolecule metabolic process | 50 | 16.4 | 1.00E-04 | 3.20E-02 |
| GOTERM_BP_ALL | cellular process | 88 | 28.9 | 1.40E-04 | 3.30E-02 |
| GOTERM_MF_ALL | binding | 127 | 41.6 | 2.60E-04 | 7.90E-02 |
| GOTERM_BP_ALL | regulation of RNA metabolic process | 24 | 7.9 | 3.10E-04 | 5.90E-02 |
| GOTERM_BP_ALL | regulation of gene expression | 31 | 10.2 | 3.60E-04 | 5.60E-02 |
| GOTERM_BP_ALL | regulation of transcription | 29 | 9.5 | 3.80E-04 | 5.10E-02 |
| GOTERM_BP_ALL | regulation of nucleobase. nucleoside. nucleotide and nucleic acid metabolic process | 30 | 9.8 | 4.10E-04 | 4.80E-02 |

^1^False Discovery rate adjusted P-value.

**Table G.** Top informative DAVID ALL categories including Gene Ontology (GO) biological processes (BP), molecular function (MF), and KEGG pathways enriched among all transcripts negatively and positively correlated with receptor (chemosensory) transporter protein 4 (*RTP4*).

| Category | Term | Count | % | P-value | FDR P-value^1^ |
| --- | --- | --- | --- | --- | --- |
| Negatively correlated | | | | | |
| GOTERM_BP_ALL | cellular metabolic process | 111 | 27.9 | 2.40E-08 | 2.80E-05 |
| GOTERM_BP_ALL | glucose metabolic process | 14 | 3.5 | 6.90E-08 | 4.00E-05 |
| GOTERM_BP_ALL | catabolic process | 34 | 8.5 | 4.60E-07 | 1.80E-04 |
| GOTERM_BP_ALL | cellular process | 151 | 37.9 | 7.10E-07 | 2.10E-04 |
| GOTERM_BP_ALL | hexose metabolic process | 14 | 3.5 | 7.20E-07 | 1.70E-04 |
| GOTERM_BP_ALL | metabolic process | 128 | 32.2 | 1.10E-06 | 2.10E-04 |
| GOTERM_BP_ALL | monosaccharide metabolic process | 14 | 3.5 | 2.30E-06 | 3.80E-04 |
| GOTERM_MF_ALL | binding | 208 | 52.3 | 2.30E-06 | 1.00E-03 |
| GOTERM_MF_ALL | protein binding | 123 | 30.9 | 1.10E-05 | 2.50E-03 |
| GOTERM_BP_ALL | macromolecule catabolic process | 21 | 5.3 | 2.30E-05 | 3.30E-03 |
| KEGG_PATHWAY | Starch and sucrose metabolism | 6 | 1.5 | 7.70E-04 | 8.60E-02 |
| KEGG_PATHWAY | Purine metabolism | 11 | 2.8 | 1.10E-03 | 6.50E-02 |
| Positively correlated | | | | | |
| GOTERM_BP_ALL | cellular process | 434 | 33.7 | 2.90E-10 | 6.20E-07 |
| GOTERM_MF_ALL | protein binding | 352 | 27.3 | 4.70E-10 | 3.40E-07 |
| GOTERM_MF_ALL | binding | 598 | 46.4 | 6.40E-08 | 2.40E-05 |
| GOTERM_BP_ALL | establishment of protein localization | 53 | 4.1 | 2.10E-06 | 9.10E-04 |
| GOTERM_BP_ALL | cellular metabolic process | 278 | 21.6 | 1.20E-06 | 1.30E-03 |
| GOTERM_BP_ALL | protein localization | 57 | 4.4 | 1.90E-06 | 1.30E-03 |
| GOTERM_BP_ALL | protein transport | 53 | 4.1 | 2.00E-06 | 1.10E-03 |
| GOTERM_BP_ALL | macromolecule localization | 64 | 5 | 5.70E-06 | 2.10E-03 |
| GOTERM_BP_ALL | cellular catabolic process | 53 | 4.1 | 4.10E-05 | 1.20E-02 |
| GOTERM_BP_ALL | cellular macromolecule metabolic process | 202 | 15.7 | 9.90E-05 | 2.60E-02 |
| KEGG_PATHWAY | Proteasome | 11 | 0.9 | 3.70E-04 | 5.90E-02 |

^1^False Discovery rate adjusted P-value.

**Table H.** Top informative DAVID ALL categories including Gene Ontology (GO) biological processes (BP) and molecular function (MF) enriched among all transcripts negatively and positively correlated with 5'-nucleotidase, cytosolic II (*NT5C2*).

| Category | Term | Count | % | P-value | FDR P-value^1^ |
| --- | --- | --- | --- | --- | --- |
| Negatively correlated | | | | | |
| GOTERM_BP_ALL | cellular metabolic process | 231 | 24.8 | 8.30E-13 | 1.50E-09 |
| GOTERM_BP_ALL | cellular process | 328 | 35.3 | 1.80E-12 | 1.60E-09 |
| GOTERM_BP_ALL | cellular macromolecule metabolic process | 175 | 18.8 | 2.00E-10 | 1.20E-07 |
| GOTERM_BP_ALL | primary metabolic process | 234 | 25.2 | 1.10E-09 | 4.70E-07 |
| GOTERM_BP_ALL | metabolic process | 266 | 28.6 | 5.00E-09 | 1.80E-06 |
| GOTERM_BP_ALL | macromolecule metabolic process | 189 | 20.3 | 2.80E-08 | 8.30E-06 |
| GOTERM_BP_ALL | cellular protein metabolic process | 111 | 11.9 | 8.90E-08 | 2.30E-05 |
| GOTERM_BP_ALL | cellular catabolic process | 47 | 5.1 | 4.40E-07 | 9.80E-05 |
| GOTERM_MF_ALL | protein binding | 250 | 26.9 | 2.30E-07 | 1.40E-04 |
| GOTERM_BP_ALL | protein transport | 42 | 4.5 | 2.40E-06 | 4.80E-04 |
| Positively correlated | | | | | |
| GOTERM_CC_ALL | intracellular | 100 | 33.4 | 3.20E-04 | 5.20E-02 |

^1^False Discovery rate adjusted P-value.

**Table I.** Top informative DAVID ALL categories including Gene Ontology (GO) biological processes (BP), molecular function (MF), and KEGG pathways enriched among all transcripts negatively and positively correlated with growth factor receptor-bound protein 10 (*GRB10*).

| Category | Term | Count | % | P-value | FDR P-value^1^ |
| --- | --- | --- | --- | --- | --- |
| Negatively correlated | | | | | |
| GOTERM_BP_ALL | cellular process | 489 | 35.2 | 4.10E-16 | 9.60E-13 |
| GOTERM_BP_ALL | cellular metabolic process | 328 | 23.6 | 6.10E-13 | 6.60E-10 |
| GOTERM_MF_ALL | protein binding | 384 | 27.7 | 8.60E-12 | 6.50E-09 |
| GOTERM_BP_ALL | cellular macromolecule metabolic process | 243 | 17.5 | 1.10E-09 | 7.70E-07 |
| GOTERM_BP_ALL | primary metabolic process | 333 | 24 | 3.10E-09 | 1.70E-06 |
| GOTERM_BP_ALL | metabolic process | 384 | 27.7 | 6.30E-09 | 2.70E-06 |
| GOTERM_BP_ALL | protein transport | 60 | 4.3 | 7.30E-08 | 2.60E-05 |
| GOTERM_BP_ALL | establishment of protein localization | 60 | 4.3 | 7.90E-08 | 2.40E-05 |
| GOTERM_BP_ALL | protein localization | 64 | 4.6 | 9.90E-08 | 2.70E-05 |
| GOTERM_BP_ALL | macromolecule localization | 73 | 5.3 | 1.40E-07 | 3.40E-05 |
| KEGG_PATHWAY | Proteasome | 11 | 0.8 | 5.90E-04 | 9.50E-02 |
| KEGG_PATHWAY | Pyrimidine metabolism | 16 | 1.2 | 1.10E-03 | 8.90E-02 |
| Positively correlated | | | | | |
| GOTERM_MF_ALL | binding | 586 | 41.7 | 1.50E-15 | 1.00E-12 |
| GOTERM_BP_ALL | cellular macromolecule metabolic process | 219 | 15.6 | 1.40E-12 | 2.80E-09 |
| GOTERM_BP_ALL | cellular process | 403 | 28.7 | 1.50E-12 | 1.50E-09 |
| GOTERM_BP_ALL | post-translational protein modification | 90 | 6.4 | 2.90E-11 | 2.00E-08 |
| GOTERM_BP_ALL | regulation of cellular metabolic process | 147 | 10.5 | 5.30E-11 | 2.70E-08 |
| GOTERM_BP_ALL | cellular metabolic process | 273 | 19.4 | 5.60E-11 | 2.30E-08 |
| GOTERM_BP_ALL | regulation of primary metabolic process | 142 | 10.1 | 9.40E-11 | 3.20E-08 |
| GOTERM_BP_ALL | biopolymer modification | 103 | 7.3 | 1.60E-10 | 4.70E-08 |
| GOTERM_BP_ALL | regulation of metabolic process | 152 | 10.8 | 1.70E-10 | 4.40E-08 |
| GOTERM_BP_ALL | regulation of biological process | 256 | 18.2 | 2.50E-10 | 5.70E-08 |
| KEGG_PATHWAY | Endocytosis | 28 | 2 | 3.30E-05 | 5.00E-03 |
| KEGG_PATHWAY | Ubiquitin mediated proteolysis | 23 | 1.6 | 5.00E-05 | 3.70E-03 |
| KEGG_PATHWAY | TGF-beta signaling pathway | 16 | 1.1 | 1.50E-04 | 7.60E-03 |
| KEGG_PATHWAY | Pathways in cancer | 34 | 2.4 | 2.50E-03 | 8.80E-02 |

^1^False Discovery rate adjusted P-value.

**Table J.** Top informative DAVID ALL categories including Gene Ontology (GO) biological processes (BP), molecular function (MF), and KEGG pathways enriched among all transcripts negatively and positively correlated with HECT domain containing E3 ubiquitin protein ligase 4 (*HECTD4*).

| Category | Term | Count | % | P-value | FDR P-value^1^ |
| --- | --- | --- | --- | --- | --- |
| Negatively correlated | | | | | |
| GOTERM_BP_ALL | cellular process | 302 | 36.3 | 2.30E-09 | 4.10E-06 |
| GOTERM_MF_ALL | protein binding | 249 | 29.9 | 4.80E-09 | 2.90E-06 |
| GOTERM_BP_ALL | cellular metabolic process | 201 | 24.2 | 1.80E-07 | 1.70E-04 |
| GOTERM_BP_ALL | metabolic process | 239 | 28.7 | 8.80E-06 | 5.30E-03 |
| GOTERM_BP_ALL | cellular macromolecule metabolic process | 146 | 17.5 | 3.50E-05 | 1.50E-02 |
| GOTERM_BP_ALL | primary metabolic process | 202 | 24.3 | 5.50E-05 | 2.00E-02 |
| GOTERM_BP_ALL | translation | 29 | 3.5 | 6.30E-05 | 1.80E-02 |
| GOTERM_BP_ALL | cellular protein metabolic process | 95 | 11.4 | 6.80E-05 | 1.70E-02 |
| GOTERM_BP_ALL | macromolecule localization | 44 | 5.3 | 1.60E-04 | 3.40E-02 |
| KEGG_PATHWAY | Proteasome | 10 | 1.2 | 8.90E-05 | 1.30E-02 |
| Positively correlated | | | | | |
| GOTERM_BP_ALL | cellular macromolecule metabolic process | 88 | 15 | 1.70E-08 | 1.80E-05 |
| GOTERM_BP_ALL | cellular process | 152 | 26 | 3.50E-08 | 1.80E-05 |
| GOTERM_BP_ALL | cellular metabolic process | 108 | 18.5 | 6.50E-08 | 2.20E-05 |
| GOTERM_MF_ALL | binding | 219 | 37.4 | 1.80E-07 | 7.30E-05 |
| GOTERM_BP_ALL | macromolecule metabolic process | 90 | 15.4 | 7.60E-06 | 1.90E-03 |
| GOTERM_MF_ALL | zinc ion binding | 57 | 9.7 | 8.60E-06 | 1.80E-03 |
| GOTERM_BP_ALL | nucleobase nucleoside nucleotide and nucleic acid metabolic process | 50 | 8.5 | 9.60E-06 | 2.00E-03 |
| GOTERM_BP_ALL | cellular nitrogen compound metabolic process | 54 | 9.2 | 1.20E-05 | 2.00E-03 |
| GOTERM_MF_ALL | transition metal ion binding | 65 | 11.1 | 2.50E-05 | 3.50E-03 |
| KEGG_PATHWAY | Spliceosome | 12 | 2.1 | 1.70E-04 | 1.80E-02 |

^1^False Discovery rate adjusted P-value.

**Table K.** Top informative DAVID ALL categories including Gene Ontology (GO) biological processes (BP), molecular function (MF), and KEGG pathways enriched among all transcripts positively correlated with AKT interacting protein (*AKTIP*).

| Category | Term | Count | % | P-value | FDR P-value^1^ |
| --- | --- | --- | --- | --- | --- |
| Positively correlated | | | | | |
| GOTERM_BP_ALL | cellular process | 220 | 35.4 | 1.50E-08 | 2.10E-05 |
| GOTERM_MF_ALL | binding | 287 | 46.1 | 1.70E-07 | 8.20E-05 |
| GOTERM_BP_ALL | cellular metabolic process | 149 | 24 | 3.80E-07 | 2.70E-04 |
| GOTERM_MF_ALL | nucleotide binding | 83 | 13.3 | 1.70E-06 | 4.10E-04 |
| GOTERM_MF_ALL | catalytic activity | 155 | 24.9 | 3.50E-05 | 5.70E-03 |
| GOTERM_MF_ALL | ligase activity | 20 | 3.2 | 7.70E-05 | 9.50E-03 |
| GOTERM_MF_ALL | purine nucleotide binding | 67 | 10.8 | 9.80E-05 | 9.60E-03 |
| GOTERM_BP_ALL | primary metabolic process | 148 | 23.8 | 9.90E-05 | 3.40E-02 |
| GOTERM_BP_ALL | metabolic process | 172 | 27.7 | 7.00E-05 | 3.20E-02 |
| KEGG_PATHWAY | Alanine, aspartate, glutamate metabolism | 7 | 1.1 | 2.30E-04 | 3.20E-02 |

^1^False Discovery rate adjusted P-value.

**Table L.** Top informative DAVID ALL categories including Gene Ontology (GO) biological processes (BP), molecular function (MF), and KEGG pathways enriched among all transcripts negatively and positively correlated with alpha-kinase 3 (*ALPK3*).

| Category | Term | Count | % | P-value | FDR P-value^1^ |
| --- | --- | --- | --- | --- | --- |
| Negatively correlated | | | | | |
| GOTERM_BP_ALL | cellular metabolic process | 247 | 24.2 | 5.60E-10 | 1.10E-06 |
| GOTERM_BP_ALL | cellular process | 358 | 35.1 | 1.40E-09 | 1.40E-06 |
| GOTERM_BP_ALL | metabolic process | 299 | 29.3 | 3.60E-09 | 2.40E-06 |
| GOTERM_BP_ALL | primary metabolic process | 251 | 24.6 | 2.80E-07 | 1.40E-04 |
| GOTERM_MF_ALL | protein binding | 278 | 27.2 | 4.60E-07 | 3.00E-04 |
| KEGG_PATHWAY | Proteasome | 13 | 1.3 | 3.90E-06 | 6.50E-04 |
| GOTERM_MF_ALL | catalytic activity | 171 | 16.7 | 4.10E-05 | 1.60E-02 |
| GOTERM_BP_ALL | cellular macromolecule metabolic process | 108 | 10.6 | 4.80E-05 | 1.60E-02 |
| GOTERM_BP_ALL | cellular biosynthetic process | 86 | 8.4 | 1.10E-04 | 3.00E-02 |
| GOTERM_BP_ALL | gene expression | 78 | 7.6 | 2.60E-04 | 6.20E-02 |
| GOTERM_BP_ALL | cellular macromolecule biosynthetic process | 171 | 16.7 | 4.10E-05 | 1.60E-02 |
| Positively correlated | | | | | |
| GOTERM_MF_ALL | binding | 331 | 38.5 | 2.10E-08 | 1.10E-05 |
| GOTERM_MF_ALL | zinc ion binding | 85 | 9.9 | 2.10E-07 | 5.70E-05 |
| GOTERM_BP_ALL | regulation of cellular process | 137 | 15.9 | 1.10E-07 | 1.40E-04 |
| GOTERM_BP_ALL | regulation of biological process | 142 | 16.5 | 2.50E-07 | 1.60E-04 |
| GOTERM_MF_ALL | cation binding | 128 | 14.9 | 6.00E-07 | 1.10E-04 |
| GOTERM_MF_ALL | metal ion binding | 126 | 14.7 | 9.80E-07 | 1.30E-04 |
| GOTERM_MF_ALL | ion binding | 128 | 14.9 | 1.00E-06 | 1.10E-04 |
| GOTERM_MF_ALL | transition metal ion binding | 97 | 11.3 | 1.10E-06 | 1.00E-04 |
| GOTERM_BP_ALL | biological regulation | 146 | 17 | 2.30E-06 | 9.90E-04 |
| GOTERM_BP_ALL | cellular macromolecule metabolic process | 113 | 13.2 | 4.30E-06 | 1.40E-03 |
| KEGG_PATHWAY | Adipocytokine signaling pathway | 10 | 1.2 | 5.30E-04 | 6.70E-02 |

^1^False Discovery rate adjusted P-value.

**Table M.** Top informative DAVID ALL categories including Gene Ontology (GO) biological processes (BP), molecular function (MF), and KEGG pathways enriched among all transcripts negatively and positively correlated with transmembrane BAX inhibitor motif containing 4 (*TMBIM4*).

| Category | Term | Count | % | P-value | FDR P-value^1^ |
| --- | --- | --- | --- | --- | --- |
| Negatively correlated | | | | | |
| GOTERM_BP_ALL | cellular process | 651 | 33.3 | 1.10E-18 | 2.80E-15 |
| GOTERM_BP_ALL | cellular metabolic process | 433 | 22.2 | 1.50E-14 | 1.90E-11 |
| GOTERM_BP_ALL | cellular macromolecule metabolic process | 324 | 16.6 | 1.10E-11 | 8.80E-09 |
| GOTERM_BP_ALL | metabolic process | 519 | 26.6 | 1.60E-11 | 1.00E-08 |
| GOTERM_BP_ALL | primary metabolic process | 445 | 22.8 | 6.00E-11 | 3.00E-08 |
| GOTERM_MF_ALL | binding | 864 | 44.2 | 8.30E-10 | 7.30E-07 |
| GOTERM_MF_ALL | protein binding | 486 | 24.9 | 2.60E-09 | 1.10E-06 |
| GOTERM_BP_ALL | cellular protein metabolic process | 201 | 10.3 | 2.10E-08 | 8.80E-06 |
| GOTERM_BP_ALL | macromolecule metabolic process | 350 | 17.9 | 4.50E-08 | 1.60E-05 |
| GOTERM_BP_ALL | protein localization | 77 | 3.9 | 5.30E-07 | 1.70E-04 |
| Positively correlated | | | | | |
| GOTERM_MF_ALL | binding | 786 | 49.2 | 8.60E-27 | 7.00E-24 |
| GOTERM_BP_ALL | cellular process | 560 | 35 | 3.10E-23 | 7.60E-20 |
| GOTERM_MF_ALL | protein binding | 470 | 29.4 | 1.50E-22 | 6.10E-20 |
| GOTERM_BP_ALL | cellular macromolecule metabolic process | 295 | 18.4 | 2.00E-17 | 2.40E-14 |
| GOTERM_BP_ALL | cellular localization | 80 | 5 | 1.10E-14 | 8.60E-12 |
| GOTERM_BP_ALL | macromolecule metabolic process | 319 | 19.9 | 1.80E-13 | 1.10E-10 |
| GOTERM_BP_ALL | cellular protein metabolic process | 189 | 11.8 | 3.80E-13 | 1.80E-10 |
| GOTERM_BP_ALL | establishment of localization in cell | 72 | 4.5 | 1.50E-12 | 6.10E-10 |
| GOTERM_BP_ALL | protein localization | 80 | 5 | 5.00E-12 | 1.70E-09 |
| Positively correlated - Top 10 KEGG Pathways | | | | | |
| KEGG_PATHWAY | Ubiquitin mediated proteolysis | 29 | 1.8 | 2.60E-06 | 4.50E-04 |
| KEGG_PATHWAY | Neurotrophin signaling pathway | 26 | 1.6 | 7.10E-06 | 6.00E-04 |
| KEGG_PATHWAY | Chronic myeloid leukemia | 19 | 1.2 | 2.00E-05 | 1.10E-03 |
| KEGG_PATHWAY | Pathways in cancer | 46 | 2.9 | 4.90E-05 | 2.10E-03 |
| KEGG_PATHWAY | Aminoacyl-tRNA biosynthesis | 12 | 0.8 | 2.60E-04 | 8.80E-03 |
| KEGG_PATHWAY | Pancreatic cancer | 16 | 1 | 2.60E-04 | 7.30E-03 |
| KEGG_PATHWAY | Colorectal cancer | 18 | 1.1 | 3.80E-04 | 9.00E-03 |
| KEGG_PATHWAY | Renal cell carcinoma | 15 | 0.9 | 6.40E-04 | 1.30E-02 |
| KEGG_PATHWAY | Proteasome | 12 | 0.8 | 6.40E-04 | 1.20E-02 |
| KEGG_PATHWAY | Focal adhesion | 29 | 1.8 | 7.80E-04 | 1.30E-02 |

^1^False Discovery rate adjusted P-value.

**Table N.** Top informative DAVID ALL categories including Gene Ontology (GO) biological processes (BP), molecular function (MF), and KEGG pathways enriched among all transcripts negatively and positively correlated with syntaxin binding protein 6 (amisyn) (*STXBP6*).

| Category | Term | Count | % | P-value | FDR P-value^1^ |
| --- | --- | --- | --- | --- | --- |
| Negatively correlated | | | | | |
| GOTERM_BP_ALL | cellular process | 579 | 33.2 | 8.60E-16 | 2.20E-12 |
| GOTERM_BP_ALL | cellular metabolic process | 394 | 22.6 | 8.30E-15 | 1.00E-11 |
| GOTERM_BP_ALL | cellular macromolecule metabolic process | 297 | 17 | 2.80E-12 | 2.20E-09 |
| GOTERM_BP_ALL | metabolic process | 469 | 26.9 | 1.60E-11 | 9.60E-09 |
| GOTERM_BP_ALL | primary metabolic process | 399 | 22.9 | 3.50E-10 | 1.70E-07 |
| GOTERM_BP_ALL | cellular protein metabolic process | 187 | 10.7 | 2.60E-09 | 1.10E-06 |
| GOTERM_MF_ALL | binding | 763 | 43.8 | 3.80E-09 | 3.10E-06 |
| GOTERM_MF_ALL | protein binding | 430 | 24.7 | 1.30E-08 | 5.10E-06 |
| GOTERM_BP_ALL | macromolecule metabolic process | 317 | 18.2 | 4.30E-08 | 1.50E-05 |
| GOTERM_MF_ALL | catalytic activity | 403 | 23.1 | 4.00E-07 | 1.10E-04 |
| Positively correlated | | | | | |
| GOTERM_MF_ALL | binding | 835 | 49.3 | 3.20E-27 | 2.70E-24 |
| GOTERM_BP_ALL | cellular process | 598 | 35.3 | 2.40E-24 | 5.80E-21 |
| GOTERM_MF_ALL | protein binding | 486 | 28.7 | 8.70E-20 | 3.60E-17 |
| GOTERM_BP_ALL | cellular macromolecule metabolic process | 303 | 17.9 | 1.00E-14 | 1.20E-11 |
| GOTERM_BP_ALL | vesicle-mediated transport | 58 | 3.4 | 5.30E-12 | 4.30E-09 |
| GOTERM_BP_ALL | macromolecule localization | 95 | 5.6 | 8.20E-12 | 5.00E-09 |
| GOTERM_BP_ALL | cellular localization | 77 | 4.5 | 9.60E-12 | 4.70E-09 |
| GOTERM_BP_ALL | protein localization | 82 | 4.8 | 2.40E-11 | 9.90E-09 |
| GOTERM_BP_ALL | macromolecule metabolic process | 328 | 19.4 | 3.50E-11 | 1.20E-08 |
| GOTERM_BP_ALL | cellular metabolic process | 373 | 22 | 4.80E-11 | 1.50E-08 |
| Positively correlated - Top 10 KEGG Pathways | | | | | |
| KEGG_PATHWAY | Ubiquitin mediated proteolysis | 30 | 1.8 | 5.50E-06 | 9.50E-04 |
| KEGG_PATHWAY | Neurotrophin signaling pathway | 26 | 1.5 | 3.60E-05 | 3.10E-03 |
| KEGG_PATHWAY | Pathways in cancer | 48 | 2.8 | 1.20E-04 | 6.70E-03 |
| KEGG_PATHWAY | Fc gamma R-mediated phagocytosis | 19 | 1.1 | 5.20E-04 | 2.20E-02 |
| KEGG_PATHWAY | Chronic myeloid leukemia | 17 | 1 | 7.30E-04 | 2.50E-02 |
| KEGG_PATHWAY | Glioma | 14 | 0.8 | 2.20E-03 | 6.10E-02 |
| KEGG_PATHWAY | Aminoacyl-tRNA biosynthesis | 11 | 0.6 | 2.30E-03 | 5.50E-02 |
| KEGG_PATHWAY | Endocytosis | 29 | 1.7 | 2.50E-03 | 5.30E-02 |
| KEGG_PATHWAY | Focal adhesion | 29 | 1.7 | 3.20E-03 | 6.00E-02 |
| KEGG_PATHWAY | Proteasome | 11 | 0.6 | 4.90E-03 | 8.00E-02 |

^1^False Discovery rate adjusted P-value.

**Table O.** Top informative DAVID ALL categories including Gene Ontology (GO) biological processes (BP), molecular function (MF), and KEGG pathways enriched among all transcripts negatively correlated with X-ray repair complementing defective repair in Chinese hamster cells 2 (*XRCC2*).

| Category | Term | Count | % | P-value | p FDR P-value^1^ |
| --- | --- | --- | --- | --- | --- |
| Negatively correlated | | | | | |
| GOTERM_BP_ALL | cellular process | 614 | 34.2 | 2.80E-19 | 7.00E-16 |
| GOTERM_BP_ALL | cellular metabolic process | 413 | 23 | 9.60E-16 | 1.30E-12 |
| GOTERM_BP_ALL | cellular macromolecule metabolic process | 311 | 17.3 | 5.90E-13 | 5.00E-10 |
| GOTERM_BP_ALL | metabolic process | 491 | 27.3 | 3.40E-12 | 2.20E-09 |
| GOTERM_BP_ALL | primary metabolic process | 422 | 23.5 | 1.20E-11 | 6.30E-09 |
| GOTERM_MF_ALL | protein binding | 460 | 25.6 | 1.80E-10 | 1.50E-07 |
| GOTERM_BP_ALL | cellular protein metabolic process | 196 | 10.9 | 8.00E-10 | 3.40E-07 |
| GOTERM_MF_ALL | binding | 802 | 44.6 | 1.40E-09 | 5.80E-07 |
| GOTERM_BP_ALL | macromolecule metabolic process | 332 | 18.5 | 1.50E-08 | 5.40E-06 |
| KEGG_PATHWAY | Ribosome | 19 | 1.1 | 3.40E-04 | 5.80E-02 |

^1^False Discovery rate adjusted P-value.

**Table P.** Top informative DAVID ALL categories including Gene Ontology (GO) biological processes (BP), molecular function (MF), and KEGG pathways enriched among all transcripts negatively and positively correlated with transmembrane protein 150A (*TMEM150A*).

| Category | Term | Count | % | P-value | FDR P-value^1^ |
| --- | --- | --- | --- | --- | --- |
| Negatively correlated | | | | | |
| GOTERM_BP_ALL | cellular process | 571 | 35.9 | 2.80E-26 | 6.70E-23 |
| GOTERM_MF_ALL | binding | 764 | 48.1 | 2.20E-21 | 1.70E-18 |
| GOTERM_BP_ALL | cellular macromolecule metabolic process | 302 | 19 | 3.70E-19 | 4.40E-16 |
| GOTERM_MF_ALL | protein binding | 452 | 28.4 | 1.00E-18 | 4.00E-16 |
| GOTERM_BP_ALL | macromolecule metabolic process | 325 | 20.5 | 1.20E-14 | 9.30E-12 |
| GOTERM_BP_ALL | cellular metabolic process | 368 | 23.2 | 1.40E-14 | 8.10E-12 |
| GOTERM_BP_ALL | cellular protein metabolic process | 193 | 12.1 | 4.10E-14 | 1.90E-11 |
| GOTERM_BP_ALL | cellular localization | 76 | 4.8 | 1.10E-12 | 4.50E-10 |
| GOTERM_BP_ALL | establishment of localization in cell | 71 | 4.5 | 5.70E-12 | 1.90E-09 |
| GOTERM_BP_ALL | primary metabolic process | 378 | 23.8 | 2.40E-11 | 7.30E-09 |
| Negatively correlated - Top 10 KEGG Pathways | | | | | |
| KEGG_PATHWAY | Ubiquitin mediated proteolysis | 28 | 1.8 | 1.10E-05 | 1.80E-03 |
| KEGG_PATHWAY | Pathways in cancer | 47 | 3 | 3.30E-05 | 2.80E-03 |
| KEGG_PATHWAY | Chronic myeloid leukemia | 17 | 1.1 | 3.00E-04 | 1.70E-02 |
| KEGG_PATHWAY | Proteasome | 12 | 0.8 | 7.30E-04 | 3.00E-02 |
| KEGG_PATHWAY | Endocytosis | 29 | 1.8 | 7.50E-04 | 2.50E-02 |
| KEGG_PATHWAY | Focal adhesion | 29 | 1.8 | 9.80E-04 | 2.70E-02 |
| KEGG_PATHWAY | Neurotrophin signaling pathway | 21 | 1.3 | 1.60E-03 | 3.80E-02 |
| KEGG_PATHWAY | Toll-like receptor signaling pathway | 18 | 1.1 | 1.70E-03 | 3.50E-02 |
| KEGG_PATHWAY | Renal cell carcinoma | 14 | 0.9 | 2.30E-03 | 4.30E-02 |
| KEGG_PATHWAY | VEGF signaling pathway | 15 | 0.9 | 2.40E-03 | 4.00E-02 |
| Positively correlated | | | | | |
| GOTERM_BP_ALL | cellular process | 623 | 33.1 | 2.40E-16 | 5.50E-13 |
| GOTERM_BP_ALL | cellular metabolic process | 419 | 22.3 | 2.60E-14 | 3.20E-11 |
| GOTERM_BP_ALL | cellular macromolecule metabolic process | 316 | 16.8 | 4.60E-12 | 3.80E-09 |
| GOTERM_BP_ALL | metabolic process | 502 | 26.7 | 2.20E-11 | 1.40E-08 |
| GOTERM_MF_ALL | binding | 842 | 44.7 | 3.20E-11 | 2.70E-08 |
| GOTERM_BP_ALL | primary metabolic process | 426 | 22.6 | 6.50E-10 | 3.20E-07 |
| GOTERM_MF_ALL | protein binding | 473 | 25.1 | 1.10E-09 | 4.60E-07 |
| GOTERM_BP_ALL | macromolecule metabolic process | 339 | 18 | 4.90E-08 | 2.00E-05 |
| GOTERM_BP_ALL | cellular protein metabolic process | 193 | 10.2 | 6.30E-08 | 2.20E-05 |
| GOTERM_BP_ALL | protein localization | 74 | 3.9 | 1.10E-06 | 3.50E-04 |

^1^False Discovery rate adjusted P-value.

**Table Q.** Top informative DAVID ALL categories including Gene Ontology (GO) biological processes (BP), molecular function (MF), and KEGG pathways enriched among all transcripts negatively and positively correlated with HAUS augmin-like complex, subunit 6 (*HAUS6*).

| Category | Term | Count | % | P-value | FDR P-value^1^ |
| --- | --- | --- | --- | --- | --- |
| Negatively correlated | | | | | |
| GOTERM_BP_ALL | cellular process | 634 | 33.7 | 7.30E-18 | 1.80E-14 |
| GOTERM_BP_ALL | cellular metabolic process | 432 | 23 | 1.80E-16 | 2.80E-13 |
| GOTERM_BP_ALL | metabolic process | 515 | 27.4 | 4.50E-13 | 3.80E-10 |
| GOTERM_BP_ALL | cellular macromolecule metabolic process | 323 | 17.2 | 5.30E-13 | 3.40E-10 |
| GOTERM_BP_ALL | primary metabolic process | 439 | 23.3 | 1.20E-11 | 5.80E-09 |
| GOTERM_MF_ALL | binding | 841 | 44.7 | 2.40E-10 | 2.10E-07 |
| GOTERM_MF_ALL | protein binding | 476 | 25.3 | 6.00E-10 | 2.60E-07 |
| GOTERM_BP_ALL | cellular protein metabolic process | 200 | 10.6 | 4.50E-09 | 1.90E-06 |
| GOTERM_BP_ALL | macromolecule metabolic process | 346 | 18.4 | 1.10E-08 | 3.80E-06 |
| GOTERM_MF_ALL | catalytic activity | 438 | 23.3 | 6.00E-07 | 1.70E-04 |
| Positively correlated | | | | | |
| GOTERM_MF_ALL | binding | 776 | 45.9 | 3.00E-26 | 2.40E-23 |
| GOTERM_BP_ALL | cellular process | 547 | 32.4 | 1.40E-22 | 3.30E-19 |
| GOTERM_MF_ALL | protein binding | 449 | 26.6 | 4.70E-18 | 1.80E-15 |
| GOTERM_BP_ALL | cellular macromolecule metabolic process | 291 | 17.2 | 7.70E-18 | 9.30E-15 |
| GOTERM_BP_ALL | cellular metabolic process | 354 | 20.9 | 3.00E-13 | 2.40E-10 |
| GOTERM_BP_ALL | cellular localization | 74 | 4.4 | 2.30E-12 | 1.40E-09 |
| GOTERM_BP_ALL | macromolecule metabolic process | 306 | 18.1 | 9.30E-12 | 4.50E-09 |
| GOTERM_BP_ALL | intracellular transport | 58 | 3.4 | 9.80E-12 | 4.00E-09 |
| GOTERM_BP_ALL | protein localization | 77 | 4.6 | 3.00E-11 | 1.00E-08 |
| GOTERM_BP_ALL | cellular protein metabolic process | 179 | 10.6 | 3.30E-11 | 1.00E-08 |
| Positively correlated - Top 10 KEGG Pathways | | | | | |
| KEGG_PATHWAY | Neurotrophin signaling pathway | 27 | 1.6 | 2.30E-06 | 4.00E-04 |
| KEGG_PATHWAY | Ubiquitin mediated proteolysis | 29 | 1.7 | 2.90E-06 | 2.50E-04 |
| KEGG_PATHWAY | Toll-like receptor signaling pathway | 22 | 1.3 | 1.80E-05 | 1.00E-03 |
| KEGG_PATHWAY | Pathways in cancer | 47 | 2.8 | 2.60E-05 | 1.10E-03 |
| KEGG_PATHWAY | Renal cell carcinoma | 17 | 1 | 5.20E-05 | 1.80E-03 |
| KEGG_PATHWAY | Aminoacyl-tRNA biosynthesis | 13 | 0.8 | 5.70E-05 | 1.60E-03 |
| KEGG_PATHWAY | Pancreatic cancer | 17 | 1 | 7.70E-05 | 1.90E-03 |
| KEGG_PATHWAY | Apoptosis | 19 | 1.1 | 9.10E-05 | 1.90E-03 |
| KEGG_PATHWAY | B cell receptor signaling pathway | 17 | 1 | 1.10E-04 | 2.10E-03 |
| KEGG_PATHWAY | VEGF signaling pathway | 17 | 1 | 2.30E-04 | 3.90E-03 |

^1^False Discovery rate adjusted P-value.

**Table R.** Top informative DAVID ALL categories including Gene Ontology (GO) biological processes (BP), molecular function (MF), and KEGG pathways enriched among all transcripts negatively and positively correlated with eukaryotic translation initiation factor 2B, subunit 1 alpha, 26kDa (*EIF2B1*).

| Category | Term | Count | % | P-value | FDR P-value^1^ |
| --- | --- | --- | --- | --- | --- |
| Negatively correlated | | | | | |
| GOTERM_BP_ALL | cellular process | 667 | 33.5 | 3.60E-18 | 9.00E-15 |
| GOTERM_BP_ALL | cellular metabolic process | 436 | 21.9 | 1.20E-12 | 1.50E-09 |
| GOTERM_MF_ALL | binding | 896 | 45 | 3.80E-11 | 3.40E-08 |
| GOTERM_MF_ALL | protein binding | 507 | 25.5 | 1.30E-10 | 5.80E-08 |
| GOTERM_BP_ALL | cellular macromolecule metabolic process | 324 | 16.3 | 6.10E-10 | 5.10E-07 |
| GOTERM_BP_ALL | metabolic process | 522 | 26.2 | 2.30E-09 | 1.40E-06 |
| GOTERM_BP_ALL | primary metabolic process | 446 | 22.4 | 6.80E-09 | 3.40E-06 |
| GOTERM_BP_ALL | cellular protein metabolic process | 204 | 10.2 | 5.70E-08 | 2.40E-05 |
| GOTERM_BP_ALL | protein localization | 80 | 4 | 2.10E-07 | 7.50E-05 |
| GOTERM_BP_ALL | protein transport | 73 | 3.7 | 5.30E-07 | 1.70E-04 |
| Positively correlated | | | | | |
| GOTERM_MF_ALL | binding | 676 | 45.9 | 2.50E-20 | 1.80E-17 |
| GOTERM_BP_ALL | cellular process | 481 | 32.6 | 3.20E-20 | 7.10E-17 |
| GOTERM_BP_ALL | cellular macromolecule metabolic process | 256 | 17.4 | 1.30E-15 | 1.50E-12 |
| GOTERM_MF_ALL | protein binding | 394 | 26.7 | 2.80E-15 | 9.80E-13 |
| GOTERM_BP_ALL | cellular localization | 72 | 4.9 | 2.90E-14 | 2.20E-11 |
| GOTERM_BP_ALL | establishment of localization in cell | 67 | 4.5 | 2.60E-13 | 1.40E-10 |
| GOTERM_BP_ALL | cellular metabolic process | 316 | 21.4 | 4.70E-13 | 2.10E-10 |
| GOTERM_BP_ALL | cellular protein metabolic process | 166 | 11.3 | 1.60E-12 | 6.10E-10 |
| GOTERM_BP_ALL | macromolecule metabolic process | 272 | 18.5 | 3.10E-11 | 9.80E-09 |
| GOTERM_BP_ALL | intracellular transport | 52 | 3.5 | 7.40E-11 | 2.10E-08 |
| Positively correlated - Top 10 KEGG Pathways | | | | | |
| KEGG_PATHWAY | Chronic myeloid leukemia | 19 | 1.3 | 3.50E-06 | 5.40E-04 |
| KEGG_PATHWAY | ErbB signaling pathway | 19 | 1.3 | 7.80E-06 | 6.10E-04 |
| KEGG_PATHWAY | Neurotrophin signaling pathway | 22 | 1.5 | 9.30E-05 | 4.80E-03 |
| KEGG_PATHWAY | Insulin signaling pathway | 22 | 1.5 | 2.20E-04 | 8.40E-03 |
| KEGG_PATHWAY | Ubiquitin mediated proteolysis | 23 | 1.6 | 2.20E-04 | 6.70E-03 |
| KEGG_PATHWAY | Pathways in cancer | 40 | 2.7 | 2.50E-04 | 6.50E-03 |
| KEGG_PATHWAY | Proteasome | 11 | 0.7 | 9.60E-04 | 2.10E-02 |
| KEGG_PATHWAY | Glioma | 13 | 0.9 | 1.00E-03 | 2.00E-02 |
| KEGG_PATHWAY | Wnt signaling pathway | 22 | 1.5 | 1.30E-03 | 2.10E-02 |
| KEGG_PATHWAY | Acute myeloid leukemia | 12 | 0.8 | 1.70E-03 | 2.60E-02 |

^1^False Discovery rate adjusted P-value.

**Table S.** Top informative DAVID ALL categories including Gene Ontology (GO) biological processes (BP), molecular function (MF), and KEGG pathways enriched among all transcripts negatively and positively correlated with ectonucleotide pyrophosphatase/phosphodiesterase 4 (putative) (*ENPP4*).

| Category | Term | Count | % | P-value | FDR P-value^1^ |
| --- | --- | --- | --- | --- | --- |
| Negatively correlated | | | | | |
| GOTERM_BP_ALL | cellular process | 654 | 33.8 | 1.30E-18 | 3.30E-15 |
| GOTERM_BP_ALL | cellular metabolic process | 429 | 22.2 | 3.80E-13 | 4.90E-10 |
| GOTERM_BP_ALL | metabolic process | 519 | 26.8 | 5.10E-11 | 4.40E-08 |
| GOTERM_MF_ALL | binding | 874 | 45.2 | 2.60E-10 | 2.30E-07 |
| GOTERM_BP_ALL | cellular macromolecule metabolic process | 319 | 16.5 | 2.60E-10 | 1.70E-07 |
| GOTERM_MF_ALL | protein binding | 495 | 25.6 | 3.50E-10 | 1.60E-07 |
| GOTERM_BP_ALL | primary metabolic process | 441 | 22.8 | 9.30E-10 | 4.80E-07 |
| GOTERM_BP_ALL | cellular protein metabolic process | 201 | 10.4 | 3.30E-08 | 1.40E-05 |
| GOTERM_BP_ALL | macromolecule metabolic process | 345 | 17.8 | 5.90E-07 | 2.20E-04 |
| GOTERM_MF_ALL | catalytic activity | 452 | 23.4 | 1.30E-06 | 3.90E-04 |
| KEGG_PATHWAY | Ribosome | 20 | 1 | 2.80E-04 | 4.90E-02 |
| KEGG_PATHWAY | Proteasome | 13 | 0.7 | 6.40E-04 | 5.50E-02 |
| Positively correlated | | | | | |
| GOTERM_MF_ALL | binding | 835 | 45.4 | 1.10E-25 | 8.40E-23 |
| GOTERM_BP_ALL | cellular macromolecule metabolic process | 314 | 17.1 | 1.00E-20 | 2.40E-17 |
| GOTERM_BP_ALL | cellular process | 568 | 30.9 | 3.10E-20 | 3.70E-17 |
| GOTERM_BP_ALL | cellular metabolic process | 379 | 20.6 | 4.30E-15 | 3.40E-12 |
| GOTERM_BP_ALL | macromolecule metabolic process | 334 | 18.2 | 5.90E-15 | 3.50E-12 |
| GOTERM_MF_ALL | protein binding | 468 | 25.5 | 1.70E-14 | 6.50E-12 |
| GOTERM_BP_ALL | cellular protein metabolic process | 196 | 10.7 | 9.80E-14 | 4.70E-11 |
| GOTERM_BP_ALL | primary metabolic process | 392 | 21.3 | 2.50E-12 | 9.90E-10 |
| GOTERM_MF_ALL | nucleic acid binding | 255 | 13.9 | 8.00E-12 | 2.10E-09 |
| GOTERM_MF_ALL | nucleotide binding | 213 | 11.6 | 1.50E-10 | 2.80E-08 |
| Positively correlated - Top 10 KEGG Pathways | | | | | |
| KEGG_PATHWAY | Neurotrophin signaling pathway | 26 | 1.4 | 2.60E-05 | 4.20E-03 |
| KEGG_PATHWAY | Ubiquitin mediated proteolysis | 28 | 1.5 | 3.20E-05 | 2.60E-03 |
| KEGG_PATHWAY | Pathways in cancer | 49 | 2.7 | 3.30E-05 | 1.80E-03 |
| KEGG_PATHWAY | Chronic myeloid leukemia | 19 | 1 | 5.30E-05 | 2.20E-03 |
| KEGG_PATHWAY | Adipocytokine signaling pathway | 17 | 0.9 | 7.90E-05 | 2.60E-03 |
| KEGG_PATHWAY | Aminoacyl-tRNA biosynthesis | 13 | 0.7 | 1.10E-04 | 3.00E-03 |
| KEGG_PATHWAY | Pancreatic cancer | 17 | 0.9 | 1.70E-04 | 4.10E-03 |
| KEGG_PATHWAY | ErbB signaling pathway | 18 | 1 | 3.60E-04 | 7.30E-03 |
| KEGG_PATHWAY | Glioma | 15 | 0.8 | 5.60E-04 | 1.00E-02 |
| KEGG_PATHWAY | B cell receptor signaling pathway | 16 | 0.9 | 8.10E-04 | 1.30E-02 |

^1^False Discovery rate adjusted P-value.

**Table T.** Top informative DAVID ALL categories including Gene Ontology (GO) biological processes (BP), molecular function (MF), and KEGG pathways enriched among all transcripts negatively and positively correlated with anoctamin 1, calcium activated chloride channel (*ANO1*).

| Category | Term | Count | % | P-value | FDR P-value^1^ |
| --- | --- | --- | --- | --- | --- |
| Negatively correlated | | | | | |
| GOTERM_BP_ALL | cellular process | 536 | 34 | 3.80E-18 | 8.30E-15 |
| GOTERM_BP_ALL | cellular metabolic process | 361 | 22.9 | 1.30E-14 | 1.40E-11 |
| GOTERM_BP_ALL | cellular macromolecule metabolic process | 277 | 17.6 | 3.00E-13 | 2.20E-10 |
| GOTERM_BP_ALL | cellular protein metabolic process | 177 | 11.2 | 1.80E-10 | 1.00E-07 |
| GOTERM_BP_ALL | metabolic process | 423 | 26.8 | 3.10E-10 | 1.40E-07 |
| GOTERM_BP_ALL | primary metabolic process | 363 | 23 | 1.00E-09 | 3.80E-07 |
| GOTERM_MF_ALL | binding | 692 | 43.9 | 5.80E-09 | 4.60E-06 |
| GOTERM_BP_ALL | macromolecule metabolic process | 293 | 18.6 | 1.30E-08 | 4.10E-06 |
| GOTERM_MF_ALL | protein binding | 387 | 24.6 | 1.20E-07 | 4.70E-05 |
| GOTERM_BP_ALL | biopolymer modification | 111 | 7 | 2.40E-06 | 6.40E-04 |
| Positively correlated | | | | | |
| GOTERM_MF_ALL | binding | 860 | 48.7 | 1.10E-25 | 9.40E-23 |
| GOTERM_BP_ALL | cellular process | 611 | 34.6 | 3.30E-23 | 8.50E-20 |
| GOTERM_MF_ALL | protein binding | 495 | 28 | 4.10E-18 | 1.70E-15 |
| GOTERM_BP_ALL | cellular macromolecule metabolic process | 317 | 18 | 2.10E-16 | 2.90E-13 |
| GOTERM_BP_ALL | macromolecule metabolic process | 349 | 19.8 | 4.60E-14 | 4.00E-11 |
| GOTERM_BP_ALL | cellular metabolic process | 394 | 22.3 | 1.70E-13 | 1.10E-10 |
| GOTERM_BP_ALL | primary metabolic process | 415 | 23.5 | 2.20E-12 | 1.10E-09 |
| GOTERM_BP_ALL | protein localization | 83 | 4.7 | 4.50E-11 | 2.00E-08 |
| GOTERM_BP_ALL | cellular localization | 76 | 4.3 | 1.10E-10 | 4.10E-08 |
| GOTERM_BP_ALL | vesicle-mediated transport | 56 | 3.2 | 1.80E-10 | 5.80E-08 |
| Positively correlated - Top 10 KEGG Pathways | | | | | |
| KEGG_PATHWAY | Pathways in cancer | 50 | 2.8 | 4.70E-05 | 8.10E-03 |
| KEGG_PATHWAY | Ubiquitin mediated proteolysis | 28 | 1.6 | 6.50E-05 | 5.60E-03 |
| KEGG_PATHWAY | Neurotrophin signaling pathway | 24 | 1.4 | 3.70E-04 | 2.10E-02 |
| KEGG_PATHWAY | Spliceosome | 24 | 1.4 | 6.10E-04 | 2.60E-02 |
| KEGG_PATHWAY | Chronic myeloid leukemia | 17 | 1 | 9.00E-04 | 3.10E-02 |
| KEGG_PATHWAY | Prostate cancer | 18 | 1 | 1.40E-03 | 3.90E-02 |
| KEGG_PATHWAY | Fc gamma R-mediated phagocytosis | 18 | 1 | 1.80E-03 | 4.40E-02 |
| KEGG_PATHWAY | Adherens junction | 15 | 0.8 | 2.60E-03 | 5.60E-02 |
| KEGG_PATHWAY | Lysosome | 21 | 1.2 | 3.00E-03 | 5.70E-02 |
| KEGG_PATHWAY | Adipocytokine signaling pathway | 14 | 0.8 | 4.10E-03 | 7.00E-02 |

^1^False Discovery rate adjusted P-value.

**Table U.** Top informative DAVID ALL categories including Gene Ontology (GO) biological processes (BP), molecular function (MF), and KEGG pathways enriched among all transcripts negatively and positively correlated with solute carrier family 25, member 44 (*SLC25A44*).

| Category | Term | Count | % | P-value | FDR P-value^1^ |
| --- | --- | --- | --- | --- | --- |
| Negatively correlated | | | | | |
| GOTERM_BP_ALL | cellular process | 653 | 34.2 | 3.60E-18 | 9.10E-15 |
| GOTERM_BP_ALL | cellular metabolic process | 432 | 22.6 | 9.50E-14 | 1.20E-10 |
| GOTERM_BP_ALL | metabolic process | 520 | 27.2 | 4.10E-11 | 3.50E-08 |
| GOTERM_BP_ALL | cellular macromolecule metabolic process | 321 | 16.8 | 1.20E-10 | 7.50E-08 |
| GOTERM_MF_ALL | protein binding | 485 | 25.4 | 4.80E-10 | 4.30E-07 |
| GOTERM_BP_ALL | primary metabolic process | 442 | 23.2 | 7.30E-10 | 3.70E-07 |
| GOTERM_MF_ALL | binding | 853 | 44.7 | 1.50E-09 | 6.50E-07 |
| GOTERM_BP_ALL | cellular protein metabolic process | 201 | 10.5 | 3.60E-08 | 1.50E-05 |
| GOTERM_BP_ALL | macromolecule metabolic process | 346 | 18.1 | 4.60E-07 | 1.70E-04 |
| GOTERM_MF_ALL | catalytic activity | 444 | 23.3 | 1.10E-06 | 3.30E-04 |
| KEGG_PATHWAY | Ribosome | 20 | 1 | 2.70E-04 | 4.60E-02 |
| KEGG_PATHWAY | Proteasome | 13 | 0.7 | 6.10E-04 | 5.30E-02 |
| Positively correlated | | | | | |
| GOTERM_MF_ALL | binding | 857 | 46.2 | 7.50E-28 | 6.10E-25 |
| GOTERM_BP_ALL | cellular process | 590 | 31.8 | 9.80E-23 | 2.30E-19 |
| GOTERM_BP_ALL | cellular macromolecule metabolic process | 325 | 17.5 | 5.40E-22 | 6.30E-19 |
| GOTERM_BP_ALL | macromolecule metabolic process | 347 | 18.7 | 2.30E-16 | 1.70E-13 |
| GOTERM_BP_ALL | cellular metabolic process | 390 | 21 | 1.40E-15 | 8.40E-13 |
| GOTERM_MF_ALL | protein binding | 475 | 25.6 | 4.00E-14 | 1.60E-11 |
| GOTERM_MF_ALL | nucleic acid binding | 264 | 14.2 | 6.10E-13 | 1.70E-10 |
| GOTERM_BP_ALL | cellular protein metabolic process | 195 | 10.5 | 3.20E-12 | 1.50E-09 |
| GOTERM_BP_ALL | primary metabolic process | 400 | 21.6 | 5.80E-12 | 2.30E-09 |
| GOTERM_BP_ALL | cellular localization | 74 | 4 | 1.30E-10 | 4.40E-08 |
| Positively correlated - Top 10 KEGG Pathways | | | | | |
| KEGG_PATHWAY | Ubiquitin mediated proteolysis | 29 | 1.6 | 1.10E-05 | 1.70E-03 |
| KEGG_PATHWAY | Pathways in cancer | 49 | 2.6 | 3.20E-05 | 2.60E-03 |
| KEGG_PATHWAY | Neurotrophin signaling pathway | 25 | 1.3 | 7.20E-05 | 3.90E-03 |
| KEGG_PATHWAY | Aminoacyl-tRNA biosynthesis | 13 | 0.7 | 1.10E-04 | 4.40E-03 |
| KEGG_PATHWAY | Glioma | 16 | 0.9 | 1.60E-04 | 5.10E-03 |
| KEGG_PATHWAY | Chronic myeloid leukemia | 18 | 1 | 1.80E-04 | 4.80E-03 |
| KEGG_PATHWAY | Focal adhesion | 31 | 1.7 | 5.10E-04 | 1.20E-02 |
| KEGG_PATHWAY | Adherens junction | 16 | 0.9 | 5.70E-04 | 1.20E-02 |
| KEGG_PATHWAY | Pancreatic cancer | 16 | 0.9 | 5.70E-04 | 1.20E-02 |
| KEGG_PATHWAY | RNA degradation | 14 | 0.8 | 6.30E-04 | 1.10E-02 |

^1^False Discovery rate adjusted P-value.

**Table V.** Top informative DAVID ALL categories including Gene Ontology (GO) biological processes (BP), molecular function (MF), and KEGG pathways enriched among all transcripts negatively and positively correlated with CDK5 regulatory subunit associated protein 3 (*CDK5RAP3*).

| Category | Term | Count | % | P-value | FDR P-value^1^ |
| --- | --- | --- | --- | --- | --- |
| Negatively correlated | | | | | |
| GOTERM_BP_ALL | cellular process | 598 | 33.2 | 1.00E-15 | 2.40E-12 |
| GOTERM_BP_ALL | cellular metabolic process | 391 | 21.7 | 2.80E-11 | 3.30E-08 |
| GOTERM_MF_ALL | binding | 806 | 44.8 | 4.10E-10 | 3.50E-07 |
| GOTERM_BP_ALL | cellular macromolecule metabolic process | 296 | 16.4 | 5.20E-10 | 4.00E-07 |
| GOTERM_BP_ALL | primary metabolic process | 407 | 22.6 | 3.20E-09 | 1.90E-06 |
| GOTERM_BP_ALL | metabolic process | 469 | 26.1 | 1.60E-08 | 7.60E-06 |
| GOTERM_MF_ALL | protein binding | 449 | 24.9 | 2.10E-08 | 9.10E-06 |
| GOTERM_BP_ALL | cellular protein metabolic process | 184 | 10.2 | 2.20E-07 | 8.60E-05 |
| GOTERM_BP_ALL | macromolecule metabolic process | 320 | 17.8 | 7.40E-07 | 2.50E-04 |
| GOTERM_BP_ALL | protein transport | 64 | 3.6 | 7.30E-06 | 2.20E-03 |
| Positively correlated | | | | | |
| GOTERM_BP_ALL | cellular process | 568 | 35.4 | 1.10E-24 | 2.60E-21 |
| GOTERM_MF_ALL | binding | 764 | 47.6 | 8.90E-20 | 7.20E-17 |
| GOTERM_BP_ALL | cellular macromolecule metabolic process | 294 | 18.3 | 2.50E-16 | 2.60E-13 |
| GOTERM_MF_ALL | protein binding | 442 | 27.5 | 3.30E-15 | 1.30E-12 |
| GOTERM_BP_ALL | macromolecule metabolic process | 321 | 20 | 1.90E-13 | 1.50E-10 |
| GOTERM_BP_ALL | cellular metabolic process | 362 | 22.6 | 6.80E-13 | 4.00E-10 |
| GOTERM_BP_ALL | cellular localization | 75 | 4.7 | 3.70E-12 | 1.70E-09 |
| GOTERM_BP_ALL | vesicle-mediated transport | 56 | 3.5 | 5.20E-12 | 2.00E-09 |
| GOTERM_BP_ALL | cellular protein metabolic process | 185 | 11.5 | 1.20E-11 | 3.90E-09 |
| GOTERM_BP_ALL | primary metabolic process | 378 | 23.6 | 3.80E-11 | 1.10E-08 |
| Positively correlated - Top 10 KEGG Pathways | | | | | |
| KEGG_PATHWAY | Proteasome | 16 | 1 | 1.30E-06 | 2.20E-04 |
| KEGG_PATHWAY | Ubiquitin mediated proteolysis | 27 | 1.7 | 3.30E-05 | 2.90E-03 |
| KEGG_PATHWAY | Aminoacyl-tRNA biosynthesis | 13 | 0.8 | 6.40E-05 | 3.70E-03 |
| KEGG_PATHWAY | Renal cell carcinoma | 15 | 0.9 | 7.50E-04 | 3.20E-02 |
| KEGG_PATHWAY | Chronic myeloid leukemia | 16 | 1 | 9.70E-04 | 3.30E-02 |
| KEGG_PATHWAY | Spliceosome | 22 | 1.4 | 1.00E-03 | 2.90E-02 |
| KEGG_PATHWAY | Neurotrophin signaling pathway | 21 | 1.3 | 1.60E-03 | 4.00E-02 |
| KEGG_PATHWAY | ErbB signaling pathway | 16 | 1 | 1.70E-03 | 3.60E-02 |
| KEGG_PATHWAY | Insulin signaling pathway | 21 | 1.3 | 3.30E-03 | 6.20E-02 |
| KEGG_PATHWAY | Endocytosis | 27 | 1.7 | 3.50E-03 | 5.90E-02 |

^1^False Discovery rate adjusted P-value.

**Table W.** Top informative DAVID ALL categories including Gene Ontology (GO) biological processes (BP), molecular functions (MF), and cellular component (CC) enriched among the higher negative and positive values for both RIF1 and RIF2.

| **Category** | **Term** | **Count^1^** | **P-Value** | **FDR P-value^2^** |
| --- | --- | --- | --- | --- |
| **Top negative RIF1** | | | | |
| MF ALL | Binding | 1124 | 2.90E-08 | 2.90E-05 |
| BP ALL | Response to stimulus | 216 | 9.10E-07 | 2.80E-03 |
| BP ALL | Oxoacid metabolic process | 71 | 1.40E-05 | 2.20E-02 |
| BP ALL | Carboxylic acid metabolic process | 71 | 1.40E-05 | 2.20E-02 |
| BP ALL | Organic acid metabolic process | 71 | 1.60E-05 | 1.60E-02 |
| BP ALL | Cellular process | 790 | 2.00E-05 | 1.50E-02 |
| BP ALL | Cellular ketone metabolic process | 72 | 2.40E-05 | 1.50E-02 |
| BP ALL | Developmental process | 192 | 5.70E-05 | 2.90E-02 |
| BP ALL | Lipid metabolic process | 85 | 6.40E-05 | 2.70E-02 |
| BP ALL | Response to external stimulus | 65 | 1.10E-04 | 4.20E-02 |
| **Top positive RIF2** | | | | |
| CC ALL | Intracellular part | 1008 | 5.60E-44 | 2.90E-41 |
| CC ALL | Intracellular | 1096 | 6.60E-42 | 1.70E-39 |
| BP ALL | Cellular process | 1022 | 4.70E-41 | 1.50E-37 |
| CC ALL | Cytoplasm | 728 | 4.70E-37 | 8.00E-35 |
| BP ALL | Cellular metabolic process | 681 | 7.80E-30 | 1.20E-26 |
| CC ALL | Organelle | 837 | 1.20E-26 | 1.50E-24 |
| CC ALL | Intracellular organelle | 836 | 1.50E-26 | 1.50E-24 |
| CC ALL | Cytoplasmic part | 509 | 4.80E-26 | 4.10E-24 |
| CC ALL | Intracellular membrane-bounded organelle | 743 | 7.10E-25 | 5.10E-23 |
| CC ALL | Membrane-bounded organelle | 744 | 7.40E-25 | 4.70E-23 |

^1^ Number of genes in the enriched category.

^2^ False Discovery rate adjusted P-value.

**Table X**. Top informative DAVID ALL categories including Gene Ontology (GO) biological processes (BP) and cellular component (CC) enriched for all Phenotypic Impact factor (PIF) scores.

| **Category** | **Term** | **Count^1^** | **P-Value** | **FDR P-value^2^** |
| --- | --- | --- | --- | --- |
| BP ALL | Cellular metabolic process | 186 | 4.90E-08 | 8.30E-05 |
| BP ALL | Cellular process | 267 | 3.00E-07 | 2.60E-04 |
| BP ALL | Metabolic process | 214 | 4.30E-05 | 2.40E-02 |
| CC ALL | Intracellular | 288 | 5.30E-05 | 1.50E-02 |
| BP ALL | Cellular biosynthetic process | 83 | 1.60E-04 | 6.40E-02 |
| BP ALL | Cellular macromolecule metabolic process | 130 | 1.70E-04 | 5.60E-02 |
| BP ALL | Cellular nitrogen compound metabolic process | 84 | 1.80E-04 | 5.00E-02 |
| BP ALL | Nitrogen compound metabolic process | 86 | 2.30E-04 | 5.40E-02 |
| CC ALL | Intracellular part | 256 | 2.60E-04 | 3.70E-02 |
| BP ALL | Gene expression | 66 | 3.60E-04 | 7.40E-02 |
| BP ALL | Biosynthetic process | 85 | 3.70E-04 | 6.70E-02 |
| BP ALL | Primary metabolic process | 179 | 4.10E-04 | 6.80E-02 |

^1^ Number of genes in the enriched category.

^2^ False Discovery rate adjusted P-value.

**Table Y.** List of 251 significant pathways identified as enriched by IPA software (p-value < 0.1) for the miRNAs *bta-mir-133a-2.*

| **Ingenuity Canonical Pathways** | **P-value** |
| --- | --- |
| Protein Kinase A Signaling | 3.98E-12 |
| Axonal Guidance Signaling | 4.47E-10 |
| HIPPO signaling | 2.57E-08 |
| Molecular Mechanisms of Cancer | 4.79E-08 |
| Role of NFAT in Cardiac Hypertrophy | 6.76E-08 |
| Dopamine-DARPP32 Feedback in cAMP Signaling | 6.76E-08 |
| Cardiac Hypertrophy Signaling | 1.05E-07 |
| ILK Signaling | 1.12E-07 |
| RhoGDI Signaling | 1.29E-07 |
| Ephrin Receptor Signaling | 1.58E-07 |
| Cardiac β-adrenergic Signaling | 3.71E-07 |
| Thrombin Signaling | 7.08E-07 |
| Paxillin Signaling | 7.08E-07 |
| p70S6K Signaling | 8.71E-07 |
| Calcium Signaling | 8.71E-07 |
| Synaptic Long Term Depression | 1.02E-06 |
| AMPK Signaling | 1.41E-06 |
| Epithelial Adherens Junction Signaling | 2.40E-06 |
| GNRH Signaling | 3.09E-06 |
| Ephrin B Signaling | 3.16E-06 |
| Phospholipase C Signaling | 3.24E-06 |
| Breast Cancer Regulation by Stathmin1 | 3.80E-06 |
| Integrin Signaling | 3.98E-06 |
| Actin Cytoskeleton Signaling | 4.90E-06 |
| PI3K/AKT Signaling | 5.89E-06 |
| PI3K Signaling in B Lymphocytes | 6.46E-06 |
| CXCR4 Signaling | 7.59E-06 |
| Cellular Effects of Sildenafil (Viagra) | 7.94E-06 |
| Signaling by Rho Family GTPases | 1.38E-05 |
| PTEN Signaling | 1.41E-05 |
| Sperm Motility | 2.57E-05 |
| RAR Activation | 3.39E-05 |
| B Cell Receptor Signaling | 3.80E-05 |
| FLT3 Signaling in Hematopoietic Progenitor Cells | 4.36E-05 |
| Tec Kinase Signaling | 4.90E-05 |
| Hepatic Fibrosis / Hepatic Stellate Cell Activation | 5.13E-05 |
| PPARα/RXRα Activation | 6.03E-05 |
| G-Protein Coupled Receptor Signaling | 6.46E-05 |
| Germ Cell-Sertoli Cell Junction Signaling | 6.76E-05 |
| TR/RXR Activation | 7.41E-05 |
| Acetate Conversion to Acetyl-CoA | 7.59E-05 |
| Role of NFAT in Regulation of the Immune Response | 8.13E-05 |
| ERK/MAPK Signaling | 9.33E-05 |
| Growth Hormone Signaling | 0.00011 |
| Role of Tissue Factor in Cancer | 0.00011 |
| α-Adrenergic Signaling | 0.00011 |
| IL-8 Signaling | 0.00012 |
| Leptin Signaling in Obesity | 0.00013 |
| Reelin Signaling in Neurons | 0.00014 |
| Gap Junction Signaling | 0.00014 |
| Fcγ Receptor-mediated Phagocytosis in Macrophages and Monocytes | 0.00015 |
| CREB Signaling in Neurons | 0.00017 |
| IL-3 Signaling | 0.00018 |
| Endothelin-1 Signaling | 0.00019 |
| Synaptic Long Term Potentiation | 0.00024 |
| Nitric Oxide Signaling in the Cardiovascular System | 0.00024 |
| FAK Signaling | 0.00030 |
| Relaxin Signaling | 0.00030 |
| CNTF Signaling | 0.00035 |
| UVA-Induced MAPK Signaling | 0.00036 |
| Gαq Signaling | 0.00041 |
| mTOR Signaling | 0.00042 |
| Melanocyte Development and Pigmentation Signaling | 0.00042 |
| GPCR-Mediated Nutrient Sensing in Enteroendocrine Cells | 0.00042 |
| Semaphorin Signaling in Neurons | 0.00046 |
| GM-CSF Signaling | 0.00049 |
| Caveolar-mediated Endocytosis Signaling | 0.00049 |
| Heme Degradation | 0.00050 |
| Rac Signaling | 0.00050 |
| Gαs Signaling | 0.00052 |
| cAMP-mediated signaling | 0.00072 |
| Corticotropin Releasing Hormone Signaling | 0.00072 |
| STAT3 Pathway | 0.00074 |
| Superpathway of Inositol Phosphate Compounds | 0.00076 |
| Calcium-induced T Lymphocyte Apoptosis | 0.00078 |
| Role of BRCA1 in DNA Damage Response | 0.00079 |
| NGF Signaling | 0.00083 |
| Agrin Interactions at Neuromuscular Junction | 0.00085 |
| G Beta Gamma Signaling | 0.00089 |
| iCOS-iCOSL Signaling in T Helper Cells | 0.00098 |
| Melatonin Signaling | 0.0010 |
| D-myo-inositol (1.4.5)-Trisphosphate Biosynthesis | 0.0011 |
| P2Y Purigenic Receptor Signaling Pathway | 0.0011 |
| Angiopoietin Signaling | 0.0012 |
| HGF Signaling | 0.0013 |
| Sertoli Cell-Sertoli Cell Junction Signaling | 0.0015 |
| ERK5 Signaling | 0.0017 |
| IGF-1 Signaling | 0.0018 |
| T Cell Receptor Signaling | 0.0018 |
| 14-3-3-mediated Signaling | 0.0018 |
| Wnt/β-catenin Signaling | 0.0019 |
| PKCθ Signaling in T Lymphocytes | 0.0021 |
| DNA Double-Strand Break Repair by Non-Homologous End Joining | 0.0022 |
| Citrulline-Nitric Oxide Cycle | 0.0022 |
| Insulin Receptor Signaling | 0.0022 |
| Colorectal Cancer Metastasis Signaling | 0.0023 |
| Renin-Angiotensin Signaling | 0.0024 |
| CDK5 Signaling | 0.0024 |
| 3-phosphoinositide Biosynthesis | 0.0026 |
| Xenobiotic Metabolism Signaling | 0.0027 |
| Regulation of Cellular Mechanics by Calpain Protease | 0.0032 |
| Clathrin-mediated Endocytosis Signaling | 0.0033 |
| IL-1 Signaling | 0.0033 |
| Diphthamide Biosynthesis | 0.0034 |
| 1D-myo-inositol Hexakisphosphate Biosynthesis V (from Ins(1.3.4)P3) | 0.0034 |
| Ephrin A Signaling | 0.0034 |
| nNOS Signaling in Skeletal Muscle Cells | 0.0035 |
| tRNA Splicing | 0.0036 |
| Gα12/13 Signaling | 0.0036 |
| JAK/Stat Signaling | 0.0037 |
| Role of Macrophages. Fibroblasts and Endothelial Cells in Rheumatoid Arthritis | 0.0040 |
| D-myo-inositol-5-phosphate Metabolism | 0.0041 |
| NF-κB Activation by Viruses | 0.0044 |
| Neuregulin Signaling | 0.0045 |
| CTLA4 Signaling in Cytotoxic T Lymphocytes | 0.0045 |
| RANK Signaling in Osteoclasts | 0.0045 |
| Regulation of eIF4 and p70S6K Signaling | 0.0051 |
| Glioblastoma Multiforme Signaling | 0.0051 |
| Regulation of the Epithelial-Mesenchymal Transition Pathway | 0.0052 |
| Mismatch Repair in Eukaryotes | 0.0055 |
| Calcium Transport I | 0.0056 |
| Leucine Degradation I | 0.0056 |
| UDP-N-acetyl-D-galactosamine Biosynthesis II | 0.0056 |
| Chondroitin and Dermatan Biosynthesis | 0.0059 |
| EGF Signaling | 0.0066 |
| Cholecystokinin/Gastrin-mediated Signaling | 0.0068 |
| RhoA Signaling | 0.0068 |
| Leukocyte Extravasation Signaling | 0.0069 |
| IL-15 Signaling | 0.0071 |
| p38 MAPK Signaling | 0.0071 |
| Role of JAK family kinases in IL-6-type Cytokine Signaling | 0.0079 |
| CD28 Signaling in T Helper Cells | 0.0079 |
| Erythropoietin Signaling | 0.0083 |
| Tight Junction Signaling | 0.0083 |
| Netrin Signaling | 0.0095 |
| Sphingosine-1-phosphate Signaling | 0.0095 |
| Aldosterone Signaling in Epithelial Cells | 0.0095 |
| Cyclins and Cell Cycle Regulation | 0.0098 |
| Prolactin Signaling | 0.0098 |
| Role of Osteoblasts. Osteoclasts and Chondrocytes in Rheumatoid Arthritis | 0.01 |
| Telomerase Signaling | 0.010 |
| Antiproliferative Role of TOB in T Cell Signaling | 0.010 |
| Virus Entry via Endocytic Pathways | 0.011 |
| Regulation of IL-2 Expression in Activated and Anergic T Lymphocytes | 0.011 |
| Isoleucine Degradation I | 0.011 |
| Phosphatidylglycerol Biosynthesis II (Non-plastidic) | 0.012 |
| Valine Degradation I | 0.012 |
| Geranylgeranyldiphosphate Biosynthesis | 0.012 |
| Androgen Signaling | 0.012 |
| CCR3 Signaling in Eosinophils | 0.013 |
| D-myo-inositol (1.4.5.6)-Tetrakisphosphate Biosynthesis | 0.013 |
| D-myo-inositol (3.4.5.6)-tetrakisphosphate Biosynthesis | 0.013 |
| autophagy | 0.013 |
| ErbB Signaling | 0.014 |
| Mechanisms of Viral Exit from Host Cells | 0.014 |
| HER-2 Signaling in Breast Cancer | 0.015 |
| GPCR-Mediated Integration of Enteroendocrine Signaling Exemplified by an L Cell | 0.015 |
| Actin Nucleation by ARP-WASP Complex | 0.015 |
| Factors Promoting Cardiogenesis in Vertebrates | 0.016 |
| VEGF Signaling | 0.016 |
| Production of Nitric Oxide and Reactive Oxygen Species in Macrophages | 0.017 |
| Acute Phase Response Signaling | 0.017 |
| Fc Epsilon RI Signaling | 0.018 |
| Chronic Myeloid Leukemia Signaling | 0.018 |
| Neurotrophin/TRK Signaling | 0.018 |
| nNOS Signaling in Neurons | 0.018 |
| Ovarian Cancer Signaling | 0.018 |
| CD27 Signaling in Lymphocytes | 0.018 |
| Endometrial Cancer Signaling | 0.018 |
| Dopamine Receptor Signaling | 0.019 |
| Type I Diabetes Mellitus Signaling | 0.020 |
| LPS-stimulated MAPK Signaling | 0.020 |
| Estrogen Receptor Signaling | 0.021 |
| Neuropathic Pain Signaling In Dorsal Horn Neurons | 0.022 |
| All-trans-decaprenyl Diphosphate Biosynthesis | 0.022 |
| S-methyl-5-thio-α-D-ribose 1-phosphate Degradation | 0.022 |
| Glioma Signaling | 0.022 |
| Huntington's Disease Signaling | 0.023 |
| Type II Diabetes Mellitus Signaling | 0.023 |
| CDP-diacylglycerol Biosynthesis I | 0.023 |
| Oncostatin M Signaling | 0.024 |
| Cleavage and Polyadenylation of Pre-mRNA | 0.024 |
| 3-phosphoinositide Degradation | 0.024 |
| Gluconeogenesis I | 0.025 |
| Ceramide Biosynthesis | 0.026 |
| Non-Small Cell Lung Cancer Signaling | 0.028 |
| Cell Cycle Regulation by BTG Family Proteins | 0.029 |
| Role of CHK Proteins in Cell Cycle Checkpoint Control | 0.029 |
| VEGF Family Ligand-Receptor Interactions | 0.029 |
| Glucocorticoid Receptor Signaling | 0.030 |
| TGF-β Signaling | 0.031 |
| T Helper Cell Differentiation | 0.031 |
| Chemokine Signaling | 0.031 |
| Mitochondrial L-carnitine Shuttle Pathway | 0.032 |
| Acute Myeloid Leukemia Signaling | 0.034 |
| 4-1BB Signaling in T Lymphocytes | 0.034 |
| Folate Transformations I | 0.034 |
| Chondroitin Sulfate Biosynthesis (Late Stages) | 0.035 |
| Adipogenesis pathway | 0.035 |
| Glioma Invasiveness Signaling | 0.039 |
| Human Embryonic Stem Cell Pluripotency | 0.40 |
| Macropinocytosis Signaling | 0.041 |
| Hereditary Breast Cancer Signaling | 0.043 |
| Role of JAK1 and JAK3 in γc Cytokine Signaling | 0.043 |
| FGF Signaling | 0.045 |
| UVB-Induced MAPK Signaling | 0.046 |
| Superpathway of Citrulline Metabolism | 0.047 |
| Regulation of Actin-based Motility by Rho | 0.047 |
| Cell Cycle: G1/S Checkpoint Regulation | 0.049 |
| fMLP Signaling in Neutrophils | 0.049 |
| G Protein Signaling Mediated by Tubby | 0.049 |
| Ketogenesis | 0.050 |
| Chondroitin Sulfate Biosynthesis | 0.052 |
| IL-4 Signaling | 0.055 |
| Gustation Pathway | 0.056 |
| IL-22 Signaling | 0.056 |
| Docosahexaenoic Acid (DHA) Signaling | 0.057 |
| Natural Killer Cell Signaling | 0.057 |
| Inhibition of Angiogenesis by TSP1 | 0.057 |
| PEDF Signaling | 0.059 |
| Thrombopoietin Signaling | 0.060 |
| Glutathione Biosynthesis | 0.060 |
| 4-aminobutyrate Degradation I | 0.060 |
| PDGF Signaling | 0.062 |
| phagosome formation | 0.063 |
| Apoptosis Signaling | 0.068 |
| Pancreatic Adenocarcinoma Signaling | 0.068 |
| Role of JAK2 in Hormone-like Cytokine Signaling | 0.068 |
| Triacylglycerol Biosynthesis | 0.068 |
| Stearate Biosynthesis I (Animals) | 0.068 |
| Glycolysis I | 0.069 |
| Sonic Hedgehog Signaling | 0.069 |
| Superpathway of Serine and Glycine Biosynthesis I | 0.074 |
| NAD Salvage Pathway III | 0.074 |
| Inositol Pyrophosphates Biosynthesis | 0.074 |
| GDP-glucose Biosynthesis | 0.074 |
| Nur77 Signaling in T Lymphocytes | 0.076 |
| Dermatan Sulfate Biosynthesis | 0.076 |
| GDNF Family Ligand-Receptor Interactions | 0.076 |
| Oxidative Ethanol Degradation III | 0.079 |
| Gαi Signaling | 0.081 |
| NF-κB Signaling | 0.083 |
| CCR5 Signaling in Macrophages | 0.085 |
| Role of MAPK Signaling in the Pathogenesis of Influenza | 0.085 |
| Phospholipases | 0.085 |
| Pyridoxal 5'-phosphate Salvage Pathway | 0.089 |
| p53 Signaling | 0.089 |
| Antioxidant Action of Vitamin C | 0.089 |
| IL-2 Signaling | 0.089 |
| BER pathway | 0.091 |
| IL-6 Signaling | 0.093 |

**Table Z.** List of 308 significant pathways identified as enriched by IPA software (p-value < 0.1) for the miRNAs *bta-mir-22.*

| **Ingenuity Canonical Pathways** | **P-value** |
| --- | --- |
| Protein Kinase A Signaling | 2E-15 |
| Molecular Mechanisms of Cancer | 1.58E-14 |
| Cardiac Hypertrophy Signaling | 7.94E-11 |
| Role of NFAT in Cardiac Hypertrophy | 5.13E-10 |
| Signaling by Rho Family GTPases | 8.51E-10 |
| CXCR4 Signaling | 2.57E-09 |
| Axonal Guidance Signaling | 3.16E-09 |
| RhoGDI Signaling | 3.31E-09 |
| ERK/MAPK Signaling | 6.46E-09 |
| GNRH Signaling | 6.61E-09 |
| IL-8 Signaling | 1.58E-08 |
| Paxillin Signaling | 1.78E-08 |
| IL-1 Signaling | 2.57E-08 |
| Ephrin Receptor Signaling | 2.82E-08 |
| ILK Signaling | 2.82E-08 |
| Tec Kinase Signaling | 4.9E-08 |
| Thrombin Signaling | 4.9E-08 |
| PTEN Signaling | 6.03E-08 |
| Actin Cytoskeleton Signaling | 7.24E-08 |
| Breast Cancer Regulation by Stathmin1 | 1.12E-07 |
| B Cell Receptor Signaling | 1.2E-07 |
| Hepatic Fibrosis / Hepatic Stellate Cell Activation | 1.41E-07 |
| Renin-Angiotensin Signaling | 1.45E-07 |
| Superpathway of Inositol Phosphate Compounds | 1.91E-07 |
| HGF Signaling | 2.63E-07 |
| Dopamine-DARPP32 Feedback in cAMP Signaling | 2.88E-07 |
| Integrin Signaling | 3.02E-07 |
| PI3K/AKT Signaling | 3.47E-07 |
| Cellular Effects of Sildenafil (Viagra) | 3.63E-07 |
| RAR Activation | 4.27E-07 |
| Cardiac β-adrenergic Signaling | 5.13E-07 |
| P2Y Purigenic Receptor Signaling Pathway | 6.31E-07 |
| Angiopoietin Signaling | 6.76E-07 |
| PPARα/RXRα Activation | 1.32E-06 |
| Ephrin B Signaling | 1.41E-06 |
| Rac Signaling | 1.45E-06 |
| p70S6K Signaling | 1.62E-06 |
| Relaxin Signaling | 2.24E-06 |
| Endothelin-1 Signaling | 2.4E-06 |
| CREB Signaling in Neurons | 3.98E-06 |
| Reelin Signaling in Neurons | 5.13E-06 |
| tRNA Charging | 5.13E-06 |
| Germ Cell-Sertoli Cell Junction Signaling | 5.75E-06 |
| Leukocyte Extravasation Signaling | 5.89E-06 |
| Sperm Motility | 7.24E-06 |
| Melanocyte Development and Pigmentation Signaling | 1.12E-05 |
| NF-κB Signaling | 1.32E-05 |
| AMPK Signaling | 1.38E-05 |
| Protein Ubiquitination Pathway | 1.55E-05 |
| G Beta Gamma Signaling | 1.66E-05 |
| T Cell Receptor Signaling | 1.74E-05 |
| Nitric Oxide Signaling in the Cardiovascular System | 1.74E-05 |
| Leptin Signaling in Obesity | 1.91E-05 |
| RhoA Signaling | 2.24E-05 |
| PAK Signaling | 2.29E-05 |
| VEGF Signaling | 2.29E-05 |
| Ketogenesis | 2.57E-05 |
| Glucocorticoid Receptor Signaling | 2.75E-05 |
| GPCR-Mediated Nutrient Sensing in Enteroendocrine Cells | 3.02E-05 |
| α-Adrenergic Signaling | 3.09E-05 |
| Phospholipase C Signaling | 3.09E-05 |
| CDK5 Signaling | 3.16E-05 |
| Semaphorin Signaling in Neurons | 3.24E-05 |
| DNA Double-Strand Break Repair by Non-Homologous End Joining | 3.39E-05 |
| Melatonin Signaling | 3.55E-05 |
| Aldosterone Signaling in Epithelial Cells | 3.55E-05 |
| Epithelial Adherens Junction Signaling | 3.89E-05 |
| Sphingosine-1-phosphate Signaling | 4.17E-05 |
| TR/RXR Activation | 4.17E-05 |
| IGF-1 Signaling | 4.27E-05 |
| 3-phosphoinositide Biosynthesis | 4.27E-05 |
| CD27 Signaling in Lymphocytes | 6.76E-05 |
| Androgen Signaling | 7.08E-05 |
| STAT3 Pathway | 0.0001 |
| Colorectal Cancer Metastasis Signaling | 0.00010 |
| D-myo-inositol-5-phosphate Metabolism | 0.00010 |
| UVA-Induced MAPK Signaling | 0.00010 |
| Production of Nitric Oxide and Reactive Oxygen Species in Macrophages | 0.00011 |
| NGF Signaling | 0.00013 |
| Role of NFAT in Regulation of the Immune Response | 0.00013 |
| Synaptic Long Term Depression | 0.00013 |
| Role of PKR in Interferon Induction and Antiviral Response | 0.00014 |
| HIPPO signaling | 0.00014 |
| Gα12/13 Signaling | 0.00015 |
| FAK Signaling | 0.00019 |
| JAK/Stat Signaling | 0.00019 |
| Role of BRCA1 in DNA Damage Response | 0.00019 |
| Insulin Receptor Signaling | 0.00019 |
| PI3K Signaling in B Lymphocytes | 0.00021 |
| RANK Signaling in Osteoclasts | 0.00024 |
| Granzyme B Signaling | 0.00024 |
| NF-κB Activation by Viruses | 0.00026 |
| BMP signaling pathway | 0.00026 |
| Gap Junction Signaling | 0.00026 |
| G-Protein Coupled Receptor Signaling | 0.00028 |
| DNA Double-Strand Break Repair by Homologous Recombination | 0.00030 |
| Netrin Signaling | 0.00030 |
| CCR3 Signaling in Eosinophils | 0.00031 |
| Huntington's Disease Signaling | 0.00035 |
| Caveolar-mediated Endocytosis Signaling | 0.00035 |
| IL-3 Signaling | 0.00035 |
| Gαq Signaling | 0.00038 |
| TGF-β Signaling | 0.00043 |
| Dopamine Receptor Signaling | 0.00046 |
| Calcium Signaling | 0.00048 |
| Synaptic Long Term Potentiation | 0.00048 |
| 3-phosphoinositide Degradation | 0.00048 |
| D-myo-inositol (1.4.5)-Trisphosphate Biosynthesis | 0.00056 |
| CNTF Signaling | 0.00059 |
| Role of CHK Proteins in Cell Cycle Checkpoint Control | 0.00060 |
| Myc Mediated Apoptosis Signaling | 0.00062 |
| 14-3-3-mediated Signaling | 0.00062 |
| Sonic Hedgehog Signaling | 0.00068 |
| Death Receptor Signaling | 0.00069 |
| Apoptosis Signaling | 0.00071 |
| PDGF Signaling | 0.00079 |
| D-myo-inositol (1.4.5.6)-Tetrakisphosphate Biosynthesis | 0.00081 |
| D-myo-inositol (3.4.5.6)-tetrakisphosphate Biosynthesis | 0.00081 |
| FLT3 Signaling in Hematopoietic Progenitor Cells | 0.00081 |
| Role of Osteoblasts. Osteoclasts and Chondrocytes in Rheumatoid Arthritis | 0.00085 |
| autophagy | 0.00087 |
| Pancreatic Adenocarcinoma Signaling | 0.00093 |
| Glutaryl-CoA Degradation | 0.0011 |
| Valine Degradation I | 0.0011 |
| SAPK/JNK Signaling | 0.0011 |
| Induction of Apoptosis by HIV1 | 0.0011 |
| Regulation of Actin-based Motility by Rho | 0.0011 |
| Leucine Degradation I | 0.0012 |
| Ketolysis | 0.0012 |
| FGF Signaling | 0.0013 |
| Tight Junction Signaling | 0.0013 |
| Regulation of the Epithelial-Mesenchymal Transition Pathway | 0.0013 |
| Cholecystokinin/Gastrin-mediated Signaling | 0.0013 |
| Xenobiotic Metabolism Signaling | 0.0013 |
| Glioma Signaling | 0.0014 |
| Sertoli Cell-Sertoli Cell Junction Signaling | 0.0014 |
| CDP-diacylglycerol Biosynthesis I | 0.0014 |
| Endoplasmic Reticulum Stress Pathway | 0.0015 |
| PKCθ Signaling in T Lymphocytes | 0.0015 |
| Pyridoxal 5'-phosphate Salvage Pathway | 0.0015 |
| Acute Myeloid Leukemia Signaling | 0.0018 |
| Superpathway of D-myo-inositol (1.4.5)-trisphosphate Metabolism | 0.0018 |
| Chronic Myeloid Leukemia Signaling | 0.0018 |
| PEDF Signaling | 0.0019 |
| Isoleucine Degradation I | 0.0019 |
| 1D-myo-inositol Hexakisphosphate Biosynthesis II (Mammalian) | 0.0020 |
| GM-CSF Signaling | 0.0020 |
| ATM Signaling | 0.0021 |
| Actin Nucleation by ARP-WASP Complex | 0.0021 |
| Role of Tissue Factor in Cancer | 0.0021 |
| CTLA4 Signaling in Cytotoxic T Lymphocytes | 0.0025 |
| Corticotropin Releasing Hormone Signaling | 0.0026 |
| p53 Signaling | 0.0026 |
| Role of Macrophages. Fibroblasts and Endothelial Cells in Rheumatoid Arthritis | 0.0027 |
| fMLP Signaling in Neutrophils | 0.0027 |
| LPS-stimulated MAPK Signaling | 0.0031 |
| ErbB Signaling | 0.0032 |
| Fc Epsilon RI Signaling | 0.0033 |
| Fcγ Receptor-mediated Phagocytosis in Macrophages and Monocytes | 0.0036 |
| Branched-chain α-keto acid Dehydrogenase Complex | 0.0037 |
| Biotin-carboxyl Carrier Protein Assembly | 0.0037 |
| Telomere Extension by Telomerase | 0.0038 |
| nNOS Signaling in Skeletal Muscle Cells | 0.0038 |
| TNFR1 Signaling | 0.0039 |
| Renal Cell Carcinoma Signaling | 0.0041 |
| Chemokine Signaling | 0.0041 |
| GPCR-Mediated Integration of Enteroendocrine Signaling Exemplified by an L Cell | 0.0041 |
| Estrogen Receptor Signaling | 0.0042 |
| Type II Diabetes Mellitus Signaling | 0.0043 |
| Ovarian Cancer Signaling | 0.0046 |
| Phosphatidylglycerol Biosynthesis II (Non-plastidic) | 0.0048 |
| D-myo-inositol (1.4.5)-trisphosphate Degradation | 0.0048 |
| Stearate Biosynthesis I (Animals) | 0.0052 |
| Agrin Interactions at Neuromuscular Junction | 0.0054 |
| Mitotic Roles of Polo-Like Kinase | 0.0056 |
| ERK5 Signaling | 0.0059 |
| Virus Entry via Endocytic Pathways | 0.0060 |
| Telomerase Signaling | 0.0060 |
| HER-2 Signaling in Breast Cancer | 0.0062 |
| VEGF Family Ligand-Receptor Interactions | 0.0062 |
| Unfolded protein response | 0.0067 |
| IL-15 Production | 0.0068 |
| Fatty Acid β-oxidation I | 0.0071 |
| Natural Killer Cell Signaling | 0.0072 |
| Adipogenesis pathway | 0.0072 |
| Wnt/β-catenin Signaling | 0.0078 |
| D-myo-inositol (1.3.4)-trisphosphate Biosynthesis | 0.0079 |
| eNOS Signaling | 0.0087 |
| GDNF Family Ligand-Receptor Interactions | 0.0089 |
| Prostanoid Biosynthesis | 0.0093 |
| Hereditary Breast Cancer Signaling | 0.0098 |
| TWEAK Signaling | 0.010 |
| Factors Promoting Cardiogenesis in Vertebrates | 0.010 |
| EGF Signaling | 0.011 |
| Growth Hormone Signaling | 0.011 |
| Mitochondrial L-carnitine Shuttle Pathway | 0.011 |
| Superpathway of Geranylgeranyldiphosphate Biosynthesis I (via Mevalonate) | 0.011 |
| Salvage Pathways of Pyrimidine Ribonucleotides | 0.013 |
| NRF2-mediated Oxidative Stress Response | 0.013 |
| Mechanisms of Viral Exit from Host Cells | 0.013 |
| Cleavage and Polyadenylation of Pre-mRNA | 0.013 |
| TCA Cycle II (Eukaryotic) | 0.013 |
| tRNA Splicing | 0.014 |
| Erythropoietin Signaling | 0.014 |
| mTOR Signaling | 0.014 |
| Lymphotoxin β Receptor Signaling | 0.014 |
| phagosome formation | 0.014 |
| Tetrahydrofolate Salvage from 5.10-methenyltetrahydrofolate | 0.015 |
| 2-oxobutanoate Degradation I | 0.015 |
| Citrulline-Nitric Oxide Cycle | 0.015 |
| Acetate Conversion to Acetyl-CoA | 0.015 |
| 1D-myo-inositol Hexakisphosphate Biosynthesis V (from Ins(1.3.4)P3) | 0.015 |
| Glioblastoma Multiforme Signaling | 0.015 |
| Ephrin A Signaling | 0.016 |
| Cyclins and Cell Cycle Regulation | 0.017 |
| Macropinocytosis Signaling | 0.017 |
| Mouse Embryonic Stem Cell Pluripotency | 0.017 |
| Docosahexaenoic Acid (DHA) Signaling | 0.018 |
| Gαs Signaling | 0.018 |
| CD40 Signaling | 0.018 |
| G Protein Signaling Mediated by Tubby | 0.019 |
| Endometrial Cancer Signaling | 0.019 |
| Tumoricidal Function of Hepatic Natural Killer Cells | 0.019 |
| Prostate Cancer Signaling | 0.019 |
| Regulation of IL-2 Expression in Activated and Anergic T Lymphocytes | 0.020 |
| CCR5 Signaling in Macrophages | 0.021 |
| p38 MAPK Signaling | 0.021 |
| IL-4 Signaling | 0.022 |
| Wnt/Ca+ pathway | 0.022 |
| Neuropathic Pain Signaling In Dorsal Horn Neurons | 0.022 |
| IL-15 Signaling | 0.022 |
| Clathrin-mediated Endocytosis Signaling | 0.022 |
| Prolactin Signaling | 0.023 |
| CD28 Signaling in T Helper Cells | 0.024 |
| Interferon Signaling | 0.025 |
| PPAR Signaling | 0.026 |
| Glucose and Glucose-1-phosphate Degradation | 0.026 |
| 4-1BB Signaling in T Lymphocytes | 0.026 |
| nNOS Signaling in Neurons | 0.026 |
| Ethanol Degradation IV | 0.026 |
| Superpathway of Cholesterol Biosynthesis | 0.026 |
| Neurotrophin/TRK Signaling | 0.027 |
| Amyotrophic Lateral Sclerosis Signaling | 0.027 |
| Hepatic Cholestasis | 0.029 |
| Cell Cycle: G1/S Checkpoint Regulation | 0.029 |
| FcγRIIB Signaling in B Lymphocytes | 0.029 |
| IL-6 Signaling | 0.029 |
| Amyloid Processing | 0.031 |
| Nucleotide Excision Repair Pathway | 0.032 |
| Non-Small Cell Lung Cancer Signaling | 0.035 |
| TNFR2 Signaling | 0.035 |
| Chondroitin and Dermatan Biosynthesis | 0.035 |
| UDP-N-acetyl-D-glucosamine Biosynthesis II | 0.035 |
| NAD Biosynthesis III | 0.035 |
| γ-linolenate Biosynthesis II (Animals) | 0.037 |
| Tryptophan Degradation III (Eukaryotic) | 0.037 |
| Acute Phase Response Signaling | 0.041 |
| Antioxidant Action of Vitamin C | 0.045 |
| UVB-Induced MAPK Signaling | 0.045 |
| IL-2 Signaling | 0.045 |
| Folate Transformations I | 0.046 |
| 2-ketoglutarate Dehydrogenase Complex | 0.047 |
| Phenylethylamine Degradation I | 0.047 |
| Methylmalonyl Pathway | 0.049 |
| Geranylgeranyldiphosphate Biosynthesis | 0.049 |
| Rapoport-Luebering Glycolytic Shunt | 0.049 |
| Glioma Invasiveness Signaling | 0.050 |
| BER pathway | 0.051 |
| Small Cell Lung Cancer Signaling | 0.052 |
| Regulation of eIF4 and p70S6K Signaling | 0.052 |
| Type I Diabetes Mellitus Signaling | 0.054 |
| Gustation Pathway | 0.055 |
| Oncostatin M Signaling | 0.055 |
| iNOS Signaling | 0.055 |
| Role of NANOG in Mammalian Embryonic Stem Cell Pluripotency | 0.060 |
| IL-17 Signaling | 0.060 |
| UDP-D-xylose and UDP-D-glucuronate Biosynthesis | 0.060 |
| All-trans-decaprenyl Diphosphate Biosynthesis | 0.060 |
| GDP-L-fucose Biosynthesis II (from L-fucose) | 0.060 |
| Formaldehyde Oxidation II (Glutathione-dependent) | 0.060 |
| Thrombopoietin Signaling | 0.064 |
| Estrogen-Dependent Breast Cancer Signaling | 0.064 |
| Role of MAPK Signaling in the Pathogenesis of Influenza | 0.064 |
| Role of JAK family kinases in IL-6-type Cytokine Signaling | 0.066 |
| Phosphatidylcholine Biosynthesis I | 0.068 |
| Acetyl-CoA Biosynthesis I (Pyruvate Dehydrogenase Complex) | 0.068 |
| Inositol Pyrophosphates Biosynthesis | 0.068 |
| Superoxide Radicals Degradation | 0.068 |
| GDP-glucose Biosynthesis | 0.068 |
| cAMP-mediated signaling | 0.068 |
| Role of JAK2 in Hormone-like Cytokine Signaling | 0.068 |
| Triacylglycerol Biosynthesis | 0.068 |
| Polyamine Regulation in Colon Cancer | 0.069 |
| Ceramide Signaling | 0.069 |
| Gαi Signaling | 0.072 |
| Dolichyl-diphosphooligosaccharide Biosynthesis | 0.074 |
| Cell Cycle: G2/M DNA Damage Checkpoint Regulation | 0.074 |
| Fatty Acid Activation | 0.076 |
| Ubiquinol-10 Biosynthesis (Eukaryotic) | 0.076 |
| Mevalonate Pathway I | 0.076 |
| Antiproliferative Role of Somatostatin Receptor 2 | 0.076 |
| VDR/RXR Activation | 0.085 |
| Assembly of RNA Polymerase II Complex | 0.087 |
| Regulation of Cellular Mechanics by Calpain Protease | 0.087 |
| Neuroprotective Role of THOP1 in Alzheimer's Disease | 0.093 |
| Role of IL-17A in Arthritis | 0.095 |
| iCOS-iCOSL Signaling in T Helper Cells | 0.095 |
| Human Embryonic Stem Cell Pluripotency | 0.097 |
